# Supplementary material for: Descriptions of the natural history of erythema nodosum leprosum to inform clinical classification – A semi-systematic review
Source: PLoS Negl Trop Dis. 2026 Mar 23;20(3):e0014100. doi: 10.1371/journal.pntd.0014100 (PMC13035235; doi:10.1371/journal.pntd.0014100)
Supplement: S1 Appendix — (PDF) [file pntd.0014100.s001.pdf]

S1 Appendix: search strategy, online resources and extracted study characteristics
Title: **Descriptions of the natural history of erythema nodosum leprosum to inform clinical classification – a semi-systematic review**

Authors: Barbara de Barros, Saba Lambert, Vivianne Lopes Antonio Dias, Diana N.J. Lockwood and Stephen L. Walker

Table A. Online resources used in the narrative review

Author(s)	Title	Source	Link
**Danielssen D.C.; Boeck W.**	*Om Spedalskhed*	National Library of Norway	https://www.nb.no/items/27f1c4d7ee099c3b1a6ea08fbf5f6da2?page=5
**Danielssen D.C.; Boeck W.**	*Traité de la spédalskhed ou éléphantiasis des Grecs* (translated from Norwegian by L.-A. Cosson)	Wellcome Collection, Public Domain Mark	https://wellcomecollection.org/works/a7x7jwdb
**Hansen G.A.; Looft C.**	*Leprosy in its Clinical and Pathological Aspects* (translated by Norman Walker)	Wellcome Collection, Public Domain Mark	https://wellcomecollection.org/works/zdsgdwx9

Box A. MEDLINE (Ovid) search strategy

1. leprosy.mp. or exp LEPROSY/
2. type 2 reaction.mp.
3. lepra reaction.mp.
4. ENL.mp.
5. Erythema Nodosum/
6. LEPROSY, BORDERLINE/
7. LEPROSY, LEPROMATOUS/
8. 1 and 2
9. 1 and 4
10. 5 or 6 or 7 or 8 or 9
11. humans.sh.
12. 10 and 11

Table B. Included studies (ordered by year)

Study	Country	Design	Definitions	Reference for definition	Comments
**Okafor, 2003 [1]**	USA	Literature review	Chronic ENL (>3 months)	None	Systematic review examining thalidomide efficacy in ENL; methodological detail limited.
**Kumar, 2004 [2]**	India	Retrospective study	Chronic ENL: continued anti-reaction treatment ≥6	None	No definition for acute ENL.

			months. Recurrent RR or ENL: recurrence >6 weeks after treatment completion.		
**Pocaterra, 2006 [3]**	India	Retrospective study	Acute ENL: single episode <6 months. Acute recurrent ENL: multiple discrete episodes. Chronic ENL: episode >6 months. Mixed acute/chronic also described.	None	Early attempt to classify ENL types; definitions not clearly operationalised.
**Van Veen, 2009 [4]**	Netherlands	Cochrane review	Acute single ENL: one episode with no recurrence during/after prednisolone. Acute multiple ENL: >1 episode. Chronic ENL: >6 months.	Pocaterra, 2006	Recurrent ENL not explicitly defined.
**Voorend, 2013 [5]**	Netherlands	Review	Single acute episodes, multiple acute episodes and chronic ENL (>6 months).	Van Veen, 2009	Based on Pocaterra classification.
**Walker, 2014 [6]**	Ethiopia	Retrospective study	Acute: single episode <24 weeks. Recurrent: ≥28 days after stopping treatment. Chronic: ≥24 weeks with continuous treatment or treatment-free period ≤27 days.	None	First clear operational definitions of acute, recurrent and chronic ENL.
**Narang, 2015 [7]**	India	Prospective pilot study	Recurrent ENL ≥6 weeks after stopping treatment; chronic ENL >24 weeks.	Pocaterra 2006; Kumar 2004	Pilot study (n=10).

**Walker, 2015 [8]**	Multicentre	Cross-sectional study	Acute <24 weeks; recurrent ≥28 days after stopping treatment; chronic ≥24 weeks with continuous treatment or treatment-free period ≤27 days.	Walker, 2014	First ENLIST study.
**Kar, 2016 [9]**	India	Prospective study	Chronic T2R: >6 months or relapse within 3 months of stopping treatment. Recurrent T2R: relapse >3 months after stopping treatment.	None	Open-label regimen comparison.
**Darlong, 2016 [10]**	India	Retrospective study	Acute ENL <24 weeks; recurrent ≥28 days after treatment; chronic >24 weeks.	Walker, 2015	Thalidomide experience study.
**Lambert, 2016 [11]**	Ethiopia	Clinical trial	New ENL: first occurrence. Recurrent ENL: recurrence after ≥4 weeks off prednisolone. Chronic ENL: episode >6 months with flare-ups during treatment.	Pocaterra, 2006	RCT comparing ciclosporin vs prednisolone.
**Nabarro, 2016 [12]**	UK	Retrospective study	Acute <6 months; recurrent ≥28 days after treatment withdrawal; chronic >6 months with continuous treatment.	None	Original reference cited incorrectly.
**Bowers, 2017 [13]**	Bangladesh	Cross-sectional study	Acute <24 weeks; recurrent ≥28 days after	Walker, 2015	HRQoL study using SF-36.

			treatment; chronic ≥24 weeks.		
**Sales, 2017 [14]**	Brazil	Cross-sectional study	Acute single episode <24 weeks; recurrent ≥28 days after treatment; chronic ≥24 weeks continuous treatment.	None	HRQoL study; ENLIST collaboration.
**Walker, 2017 [15]**	Multicentre	Score validation study	Acute <24 weeks; recurrent ≥28 days after stopping treatment; chronic ≥24 weeks continuous treatment.	Walker, 2014	ENLIST ENL Severity Scale validation.
**Negera, 2017 [16]**	Ethiopia	Case-control study	Acute <24 weeks; recurrent ≥28 days after treatment; chronic ≥24 weeks continuous treatment.	Walker, 2014	
**Maghanoy, 2017 [17]**	Philippines	Clinical trial	Repeat ENL episode defined as new lesions ≥4 weeks after previous lesions resolved or steroid discontinued.	None	Clofazimine prophylaxis trial.
**Silva Costa, 2018 [18]**	Brazil	Review	Acute <24 weeks; recurrent ≥28 days after treatment; chronic >24 weeks.	Walker, 2014	
**WHO, 2017 [19]**	–	Technical guidance	Acute <6 months; recurrent ≥28 days after treatment withdrawal; chronic >6 months	None	WHO guideline definitions.

			continuous treatment.		
**Narang, 2019 [20]**	India	Protocol	Chronic ENL ≥24 weeks; recurrent ≥28 days after treatment.	None	Protocol article.
**Neves, 2019 [21]**	Brazil	Retrospective study	Acute <24 weeks; chronic ≥24 weeks.	Walker, 2015	
**Upputuri, 2020 [22]**	India	Retrospective study	Acute <24 weeks; recurrent ≥28 days after treatment; chronic ≥24 weeks continuous treatment.	None	Thalidomide treatment study.
**de Barros, 2020 [23]**	UK	Protocol article	Acute <24 weeks; recurrent 28–84 days after treatment; chronic ≥24 weeks continuous treatment.	Walker, 2015	Clinical trial protocol.
**Cuellar-Barboza, 2020 [24]**	Mexico	Retrospective study	Chronic ENL ≥24 weeks with continuous treatment or ≤27-day treatment-free period.	Walker, 2015	Risk factor study.
**Baima de Melo, 2020 [25]**	Brazil	Cross-sectional study	Acute first episode; recurrent <24 weeks interval; chronic >24 weeks continuous treatment.	None	Epidemiological study.
**Bhat, 2020 [26]**	India	Review	Acute <24 weeks; recurrent ≥28 days after stopping treatment; chronic ≥24 weeks continuous treatment.	Walker, 2014	
**Pai, 2020 [27]**	India	Protocol record	Chronic ENL ≥24 weeks continuous	None	

			symptoms or treatment.		
**Narang, 2020 [28]**	India	Case report	Chronic ENL ≥6 months.	Kumar, 2004	
**Hanumanthu, 2021 [29]**	India	Clinical trial	Chronic ≥24 weeks; recurrent ≥28 days after treatment.	Walker, 2014	Minocycline vs clofazimine trial.
**Singla, 2021 [30]**	India	Prospective study	Acute <24 weeks; recurrent ≥28 days after stopping treatment; chronic ≥24 weeks continuous treatment.	Walker, 2015	Thalidomide study.
**Fransisca, 2021 [31]**	Indonesia	Retrospective study	Acute <6 months; chronic ≥6 months; recurrent ≥28 days after treatment withdrawal.	WHO, 2017	
**Mishra, 2022 [32]**	India	Prospective study	Recurrent ENL defined as four to seven episodes per year.	None	Treatment comparison study.
**Indrawati, 2022 [33]**	Indonesia	Retrospective study	Acute <24 weeks; recurrent ≥84 days after treatment; chronic >24 weeks continuous treatment.	De Barros, 2020	Risk factor study.
**Moura Mendes, 2022 [34]**	Brazil	Case report	Acute <24 weeks; recurrent ≥28 days after treatment; chronic ≥24 weeks continuous treatment.	Walker, 2014	
**Silva dos Santos, 2023 [35]**	Brazil	Cross-sectional study	Chronic >6 months.	None	
**Rodriguez-Riveira, 2023 [36]**	Puerto Rico	Retrospective study	Acute <24 weeks; recurrent ≥28 days after	Walker, 2014	

			treatment; chronic ≥24 weeks continuous treatment.		
**Sagar, 2024 [37]**	India	Case report	Chronic ENL with treatment-free period <27 days within previous 6 months.	None	
**Kumalasari, 2024 [38]**	Indonesia	Retrospective study	Acute <24 weeks; recurrent ≥28 days after treatment; chronic ≥24 weeks continuous treatment.	Walker, 2017	
**Mehta, 2024 [39]**	India	Review	Acute <6 months; recurrent ≥28 days after treatment; chronic >6 months continuous treatment.	None	
**Maciel-Fiuza, 2025 [40]**	Brazil	Cross-sectional study	Acute <24 weeks; recurrent ≥28 days after treatment; chronic >24 weeks continuous episodes.	None	
**Putri, 2025 [41]**	India & Indonesia	Qualitative study	Chronic ENL defined as >24 weeks with continuous treatment.	Walker, 2015	

Table C. Summary of characteristics of included studies

Study characteristics	**n (%)**
**Country**	
India*[2,3,7,9,10,22,26–30,32,37,39,42]*	**15 (36.6)**
Brazil*[14,18,21,25,34,35,40]*	**7 (17.1)**
Ethiopia*[6,11,43]*	**3 (7.3)**
Indonesia*[31,33,38]*	**3 (7.3)**
The Netherlands*[4,5]*	**2 (4.9)**
Bangladesh*[13]*	**1 (2.4)**

Mexico*[24]*	**1 (2.4)**
Puerto Rico*[36]*	**1 (2.4)**
The Philippines*[17]*	**1 (2.4)**
United Kingdom*[12]*	**1 (2.4)**
United States of America*[1]*	**1 (2.4)**
Multinational collaboration*[8,15,19,23,41]*	**5 (12.2)**
**Type of study**	
Observational cohort (retrospective)*[2,3,6,10,12,21,22,24,31,33,36,38]*	**12 (29.3)**
Cross-sectional studies*[8,13,14,25,35,40]*	**6 (14.6)**
Reviews*[1,4,5,18,26,39]*	**6 (14.6)**
Observational cohorts (prospective)*[7,9,30,32]*	**4 (9.7)**
Protocols*[23,27,42]*	**3 (7.3)**
Clinical trials*[11,17,29]*	**3 (7.3)**
Case reports*[28,34,37]*	**3 (7.3)**
Others*[15,19,41,43]*	**4 (9.7)**

References

1. Okafor MC. Thalidomide for erythema nodosum leprosum and other applications. Pharmacotherapy. 2003;23: 481–493. doi: https://dx.doi.org/10.1592/phco.23.4.481.32115
2. Kumar B, Dogra S, Kaur I. Epidemiological Characteristics of Leprosy Reactions: 15 Years Experience from North India. International Journal of Leprosy and Other Mycobacterial Diseases. 2004;72: 125. doi:10.1489/1544-581X (2004)072<0125: ECOLRY>2.0.CO;2
3. Pocaterra L, Jain S, Reddy R, Muzaffarullah S, Torres O, Suneetha S, et al. Clinical course of erythema nodosum leprosum: an 11-year cohort study in Hyderabad, India. American Journal of Tropical Medicine and Hygiene. 2006;74: 868–879. Available: http://ovidsp.ovid.com/ovidweb.cgi?T=JS&PAGE=reference&D=med6&NEWS=N&AN =16687695
4. Van Veen NHJ, Lockwood DNJ, Van Brakel WH, Ramirez J, Richardus JH. Interventions for erythema nodosum leprosum. A Cochrane review. Leprosy Review. 2009;80: 355–372. doi: 10.1002/14651858.CD006949.pub2.www.cochranelibrary.com
5. Voorend CGN, Post EB. A Systematic Review on the Epidemiological Data of Erythema Nodosum Leprosum, a Type 2 Leprosy Reaction. PLOS Neglected Tropical Diseases. 2013;7. doi: 10.1371/journal.pntd.0002440
6. Walker SL, Lebas E, Doni SN, Lockwood DNJ, Lambert SM. The Mortality Associated with Erythema Nodosum Leprosum in Ethiopia: A Retrospective Hospital-Based Study. PLOS Neglected Tropical Disease. 2014;8. doi: 10.1371/journal.pntd.0002690
7. Narang T, Sawatkar GU, Kumaran MS, Dogra S. Minocycline for Recurrent and/or Chronic Erythema Nodosum Leprosum. JAMA Dermatology. 2015;151: 1026–1028. doi: https://dx.doi.org/10.1001/jamadermatol.2015.0384
8. Walker SL, Balagon M, Darlong J, Doni SN, Hagge DA, Halwai V, et al. ENLIST 1: An International Multi-centre Cross-sectional Study of the Clinical Features of Erythema Nodosum Leprosum. Alinda M Khan D Listiawan Y BC, Group ENLIST, editors. PLOS Neglected Tropical Diseases. 2015;9: e0004065. doi: https://dx.doi.org/10.1371/journal.pntd.0004065

9. Kar HK, Gupta L. Comparative efficacy of four treatment regimens in type 2 leprosy reactions (Prednisolone alone, thalidomide alone, prednisolone plus thalidomide and prednisolone plus clofazimine). Indian Journal of Leprosy. 2016;88: 29–38.
10. Darlong J, Govindharaj P, Charles DE, Menzies A, Mani S. Experiences with Thalidomide for Erythema Nodosum Leprosum– a retrospective study. Leprosy Review. 2016;87: 211–220.
11. Lambert SM, Alembo DT, Lockwood DNJ, Nicholls PG, Idriss MH, Nigusse SD, et al. Comparison of Efficacy and Safety of Ciclosporin to Prednisolone in the Treatment of Erythema Nodosum Leprosum: Two Randomised, Double Blind, Controlled Pilot Studies in Ethiopia. PLOS Neglected Tropical Diseases. 2016;10: e0004149. doi: 10.1371/journal.pntd.0004149
12. Nabarro L, Aggarwal D, Armstrong M, Lockwood D. The use of steroids and thalidomide in the management of Erythema Nodosum Leprosum; 17 years at the Hospital for Tropical Diseases, London. Leprosy Review. 2016;87.
13. Bowers B, Butlin CR, Alam K, Lockwood DNJ, Walker SL. Health-Related Quality of Life amongst people affected by Erythema Nodosum Leprosum in Bangladesh: A Cross-sectional Study. Leprosy Review. 2017;88: 488–498.
14. Sales AM, Illarramendi X, Walker SL, Lockwood D, Sarno EN, da Costa Nery JA. The impact of erythema nodosum leprosum on health-related quality of life in Rio de Janeiro. Leprosy Review. 2017;88: 499–509.
15. Walker SL, Sales AM, Butlin CR, Shah M, Maghanoy A, Lambert SM, et al. A leprosy clinical severity scale for erythema nodosum leprosum: An international, multicentre validation study of the ENLIST ENL Severity Scale. Group ENLIST, editor. PLOS Neglected Tropical Diseases. 2017;11: e0005716. doi: https://dx.doi.org/10.1371/journal.pntd.0005716
16. Negera E, Walker SL, Bekele Y, Dockrell HM, Lockwood DN. Increased activated memory B-cells in the peripheral blood of patients with erythema nodosum leprosum reactions. PLOS Neglected Tropical Diseases. 2017;11. doi: 10.1371/journal.pntd.0006121
17. Maghanoy A, Balagon M, Saunderson P, Scheelbeek P. A prospective randomised, double-blind, placebo-controlled trial on the effect of extended clofazimine on erythema nodosum leprosum (ENL) in multibacillary (MB) leprosy. Leprosy Review. 2017.
18. Costa P do SS, Fraga LR, Kowalski TW, Daxbacher ELR, Schuler-Faccini L, Vianna FSL. Erythema Nodosum Leprosum: Update and challenges on the treatment of a neglected condition. Acta Trop. 2018;183: 134–141. doi: https://dx.doi.org/10.1016/j.actatropica.2018.02.026
19. World Health Organization, Regional Office for South-East Asia. Leprosy/Hansen Disease: Management of reactions and prevention of disabilities. Technical guidance. New Delhi; 2017. Available: http://apps.who.int/bookorders.
20. Narang T. A Study to Evaluate the Efficacy and Safety of Apremilast in Patients of Chronic and Recurrent Erythema Nodosum Leprosum – clinicaltrials.gov (NCT04822909).
21. Neves D, Sales AM, Da A, Nery C, Illarramendi X, Walker SL, et al. Retrospective study of the morbidity associated with Erythema Nodosum Leprosum in Brazilian leprosy patients. Leprosy Review. 2019; 68–77.

22. Upputuri B, Pallapati MS, Tarwater P, Srikantam A. Thalidomide in the treatment of erythema nodosum leprosum (ENL) in an outpatient setting: A five-year retrospective analysis from a leprosy referral centre in India. PLOS Neglected Tropical Diseases. 2020;14: e0008678. doi: https://dx.doi.org/10.1371/journal.pntd.0008678
23. De Barros B, Lambert SM, Shah M, Pai V V., Darlong J, Rozario BJ, et al. Methotrexate and prednisolone study in erythema nodosum leprosum (MaPs in ENL) protocol: A double-blind randomised clinical trial. BMJ Open. 2020;10: 1–7. doi:10.1136/bmjopen-2020-037700
24. Cuellar-Barboza A, Cardenas-de la Garza JA, Garcia-Lozano JA, Vera-Pineda R, Cruz-Gomez LG, Irabien-Zuniga M, et al. Leprosy reactions in North-East Mexico: epidemiology and risk factors for chronic erythema nodosum leprosum. Journal of European Academy of Dermatology and Venereology. 2020;34: e228–e229. doi: https://dx.doi.org/10.1111/jdv.16197
25. Baima de Melo C, Silva de Sa BD, Anibal Carvalho Costa F, Nunes Sarno E. Epidemiological profile and severity of erythema nodosum leprosum in Brazil: a cross-sectional study. International Journal of Dermatology. 2020;59: 856–861. doi: https://dx.doi.org/10.1111/ijd.14895
26. Bhat RM, Vaidya TP. What is New in the Pathogenesis and Management of Erythema Nodosum Leprosum. Indian Dermatology Online J. 2020;11: 482–492. doi: 10.4103/idoj.IDOJ_561_19
27. Pai V V. Treating leprosy patients at high risk of Erythema Nodosum Leprosum (ENL) reaction with additional Clofazimine. In: https://trialsearch.who.int/Trial2.aspx?TrialID=CTRI/2020/09/027702 [Internet]. 2020. Available: https://www.cochranelibrary.com/central/doi/10.1002/central/CN-02185908/full
28. Narang T, Kaushik A, Dogra S. Apremilast in chronic recalcitrant erythema nodosum leprosum: a report of two cases. British Journal of Dermatology. 2020;182: 1034–1037. doi: https://dx.doi.org/10.1111/bjd.18233
29. Hanumanthu V, Thakur V, Narang T, Dogra S. Comparison of the efficacy and safety of minocycline and clofazimine in chronic and recurrent erythema nodosum leprosum-A randomized clinical trial. Dermatology Therapy. 2021;34: e15125. doi: https://dx.doi.org/10.1111/dth.15125
30. Singla P, Joshi R, Shah BJ. Thalidomide in severe erythema nodosum leprosum (Enl)-our experience in chronic, recurrent and steroid-dependant cases. Indian Journal of Leprosy. 2021;93: 115–128. Available: http://www.ijl.org.in/2021-apr-jun/1-%20P%20Singla%20et%20al%20(115-128)%20(1).pdf
31. Fransisca C, Zulkarnain I, Ervianti E, Damayanti, Sari M, Budiono, et al. A Retrospective Study: Epidemiology, Onset, and Duration of Erythema Nodosum Leprosum in Surabaya, Indonesia. Berkala Ilmu Kesehatan Kulit dan Kelamin. 2021;33: 8–12. doi:10.20473/bikk. V33.1.2021.8-12
32. Mishra SRPK, Samal R, Behera B. Thalidomide and steroid in the management of erythema nodosum leprosum. Indian Journal of Pharmacology. 2022;54: 177–182. doi: 10.4103/ijp.ijp_946_21
33. Indrawati D, Astari L, Hidayati AN, Sawitri, Damayanti, Utomo B, et al. Risk Factors of Acute and Chronic Erythema Nodosum Leprosum in Dr. Soetomo General Academic Hospital Surabaya. Pharmacognosy Journal. 2022;14: 766–770. doi:10.5530/pj.2022.14.165

34. Mendes AFM, Gomes CM, Kurizky PS, Ianhez M. Case Report: A Case Series of Immunobiological Therapy (Anti-TNF-α) for Patients with Erythema Nodosum Leprosum. Frontiers in Medicine (Lausanne). 2022;9. doi:10.3389/fmed.2022.879527
35. Santos LSD, Drummond MR, Goulart IMB, França AFEDC, Souza EMD, Ferreira Velho PEN. Bartonella henselae as a putative trigger for chronic type 2 leprosy reactions. Brazilian Journal of Infectious Diseases. 2023;27. doi: 10.1016/j.bjid.2023.103701
36. Rodriguez-Rivera DV, Pelet-Del Toro NM, Quintero-Noriega AL. Leprosy Reactions: Experience in the Puerto Rico Hansen's Disease Population. Puerto Rico Health Science Journal. 2023;42: 197–202.
37. Sagar HK, Pawar HS. Chronic type 2 reaction in lepromatous leprosy with underlying Plasmodium falciparum infection: A case report. Tropical Doctor. 2024;54: 182–184. doi: https://dx.doi.org/10.1177/00494755231211938
38. Kumalasari DN, Listiawan MY, Prakoeswa CRS, Damayanti, Alinda MD, Utomo B. Correlation between oral infections occurrence with the severity and type of erythema nodusum leprosum in multibacilar leprosy patients in the outpatient clinic of Dr. Soetomo Hospital, Surabaya from 2017-21. Journal of Pakistan Association of Dermatologists. 2024;34: 1002–1008.
39. Mehta H, Jain S, Narang T, Chhabra S, Dogra S. Leprosy reactions: New knowledge on pathophysiology, diagnosis, treatment and prevention. Indian Journal Dermatology, Venereology and Leprology. 2024; 1–12. doi: https://dx.doi.org/10.25259/IJDVL_915_2024
40. Maciel-Fiuza MF, Sbruzzi RC, Feira MF, P.D.S.S. C, Bonamigo RR, Vettorato R, et al. Influence of Cytokine-Related genetic variants in TNF, IL6, IL1beta, and IFN gamma genes in the thalidomide treatment for Erythema nodosum leprosum in a Brazilian population sample. Human Immunology. 2025;86: 111260. doi: https://dx.doi.org/10.1016/j.humimm.2025.111260
41. Putri AI, Peters RMH, De Sabbata K, Mengistu BS, Agusni RI, Alinda MD, et al. A socio-ecological model of the management of leprosy reactions in Indonesia and India using the experiences of affected individuals, family members and healthcare providers. BMC Health Services Research. 2025;25. doi:10.1186/s12913-025-12340-5
42. Narang T. A study to evaluate the efficacy and safety of apremilast in patients of chronic and recurrent erythema nodosum leprosum. In: clinicaltrial.gov. 2019.
43. Negera E, Walker SL, Girma S, Doni SN, Tsegaye D, Lambert SM, et al. Clinico-pathological features of erythema nodosum leprosum: A case-control study at ALERT hospital, Ethiopia. PLOS Neglected Tropical Diseases. 2017;11: e0006011. doi: https://dx.doi.org/10.1371/journal.pntd.0006011
