# Supplementary material for: Descriptions of the natural history of erythema nodosum leprosum to inform clinical classification – A semi-systematic review
Source: PLoS Negl Trop Dis. 2026 Mar 23;20(3):e0014100. doi: 10.1371/journal.pntd.0014100 (PMC13035235; doi:10.1371/journal.pntd.0014100)
Supplement: S2 Appendix — (PDF) [file pntd.0014100.s002.pdf]

This article, titled *"On Erythema Nodosum Leprosum"*, was originally written in Japanese by M. Murata and published in the *Japanese Journal of Dermato-Urology* in 1912 (Issue 12, pp. 1013–1051). The article has been translated into English by Bartlemas Lionheart-Coningsby as part of a semi-systematic literature review conducted by Barbara de Barros as part of doctoral degree and funded by the Hospital and Homes of St. Giles.

On Erythema Nodosum Leprosum

M. Murata: Über Erythema nodosum leprosum.

When we observe the progression of leprosy as a whole, we encounter a variety of clinical phenomena characterised by significant variation. The appearance and disappearance of leprous eruptions is a good example of such phenomena. Eruptions that had been clearly visible may resolve naturally without any specific treatment, leaving no discernible traces. However, under certain unclear conditions and circumstances, eruptions may reappear. Regardless of severity, they may repeat the same symptoms. Fresh eruptions may also develop, leading to a more complex presentation of symptoms. Such phenomena are frequently observed as clinical features of leprosy. Leprous macules are a common symptom experienced by many leprosy patients in the early stages of the disease. These initial macules often fade after a prolonged period, with previously pigmented areas becoming almost imperceptible. However, macules are usually more pronounced when they reappear. This phenomenon is commonly observed throughout the progression of both the neural and nodular forms of leprosy. Leprous nodules and infiltrations may also spontaneously disappear or reappear, a phenomenon well recognised in clinical practice. It is important to note, however, that nodular leprosy presents a unique transient manifestation of the disease not observed in the macular or neural types. This condition, known as erythema nodosum leprosum—commonly referred to by patients as *netsukobu* (hot nodules[1])—involves the acute appearance of eruptions which cause significant suffering and often result in a critical worsening of patients' conditions.

In reviewing the literature related to this condition,[2] I was struck by the fact that the books at our disposal offer very little on the subject and contain only scant histological findings, which are summarised as follows:

Unna distinguishes three different forms of leprous nodules: (1) subcutaneous lepromata; (2) nodules appearing directly within the cutis; and (3) acute inflammatory skin nodules. He states that nodules belonging to the third form are "caused by embolism of the vessels through bacilli, appear with symptoms of fever and have their seat in the cutis and hypodermis. They resemble erythema nodosum, but are less sharply circumscribed, and are lost in inflammatory oedematous surroundings."[3] Since I have not had the opportunity to read the original text, the above is taken from a section of Scheube's book "Diseases of Warm Countries" in which Unna is cited. Although I cannot help but feel that this is a somewhat indirect approach, [this extract] presents the concept

---

[1] ねつこぶ could also be translated as "fever bumps," "fever nodules," or "hot bumps"

[2] Ambiguity in original text. Can be translated as "this disease" or "this condition" and may be referring either to leprosy in a broad sense, or more specifically to erythema nodosum leprosum. Most likely the latter.

[3] Diseases of Warm Countries, p.248, Unna

clearly. According to [Unna], nodules form as a result of inflammation. Taking the view that [these nodules] resemble erythema nodosum and are caused by bacterial embolism, [Unna] extends his theory of leprous eruptions—which he associates with lymphatic embolism—to include vascular embolism as a contributing factor in acute leprous eruptions. For instance, Sticker notes in his paper that leprous nodules and infiltrations can proliferate and thicken under various conditions. In some cases, the reemergence of nodules is accompanied by fever and joint pain, presenting symptoms akin to erythema nodosum. In other words, Sticker highlights that the appearance of leprous nodules may, in certain instances, exhibit clinical features resembling those of erythema nodosum.

Although there is no specific description of this condition in the works of Babes,[4] it is mentioned that "when leprous nodules occur in infants, they resemble erythema nodosum." Furthermore, in a section titled "Regression of Lepromata" (Rückbildung der Leprome), it is stated that if, following the regression of lepromata, fresh nodules and infiltrations develop in the same areas [affected before regression], they may be accompanied by fever (39 to 40 degrees [Centigrade]),[5] a pulse reaching 120 to 130 [BPM], headache, thirst, sensory disturbances, and frequent vertigo. Rheumatoid pain located in various joints is also reported. The affected area may exhibit erysipeloid flushing and swelling accompanied by pain. These symptoms resemble acute infectious rheumatism,[6] the cause of which [Babes] attributes in part to mixed infection and in part to the toxic irritative agents of the bacilli leprae, citing the effect of tuberculin[7] in leprosy as evidence. Although Babes' investigation was limited to a specific aspect of erythema nodosum and its eruptions in infants, and although I am unaware of what was later written [on the topic], would it not seem that the clinical manifestations associated with the reemergence of lepromata resemble those of erythema nodosum leprosum? Regardless, Babes's positing that the cause of [such symptoms] lies in leprosy's tuberculin reaction is of great interest to me. The occurrence of erysipeloid redness and swelling in the affected area during an outbreak of lepromata is also seen in the works of Scheube and Bergmann, though both authors suggest that these symptoms signify the healing or disappearance of nodular infiltrations. Scheube further notes that, following the disappearance [of lepromata], affected areas are left with residual white or dark spots. Hence, although the term 'erysipeloid redness and swelling' is used in clinical practice, it is clear that it is

[4] Victor Babeș; the accent on the "s" has been omitted so as to align with the trend seen in papers on this subject from the time, which do not use the accent when writing Babeș's name, as well as to align with the provided Japanese phonetic spelling

[5] Celcius

[6] Literal translation of the Japanese: acute infectious rheumatism [急性傳染性僂麻質斯] most likely referring to rheumatic fever (=acute rheumatism)

[7] Unusually the word 作用 is used in Japanese, lit: effect, action. This is most likely referring to the tuberculin "reaction."

not true erysipelas. It is therefore necessary to differentiate this condition from the condition forming part of my later discussion—erythema nodosum.

In the context of Japan, some years ago, Professor Yamamoto remarked on what he found to be a peculiar case of leprosy due to the eruptive conditions of the case as well as the fact that eruptions were not accompanied by sensory abnormalities. He wrote of the difficulties he experienced in using only histological findings to confirm a diagnosis of leprosy, whilst also distinguishing it from other [forms of the disease]. The histological findings in this case were, therefore, no different to those of the cutaneous leprotic changes commonly found in leprosy of the skin. To this, my mentor, the venerable Hospital Director Mitsuda, who has many years of experience and independent insight, contested Yamamoto's conjecture: "The nodules, as described by the gentleman [Yamamoto], are by no means atypical. We encounter such cases frequently. These nodules are not only induced by the administration of iodine tuberculin injections, but also in response to changes in the weather, occurring more frequently in spring and autumn. Their form resembles that of erythema nodosum. The affected area often exhibits hypersensitivity as the apex of the lesion becomes infected with pus-cocci and develops pustules, many of which gradually resolve within the space of a few weeks, leading to surface desquamation. The appearance of such nodules is often accompanied by fever." In response to this, Professor Toyama stated that: "The subject of Mr Mitsuda's research is likely something often seen in leprosy patients: erythema nodosum," thereby endorsing the notion that erythema nodosum may occur in leprosy. The appearance of nodular-like erythema in leprosy patients in Japan is a topic which has already been investigated by my seniors. The findings of their research, as presented in various papers, therefore function as a good starting point for my own paper.

Under the guidance of Hospital Director Mitsuda, I had, from September of last year to August of this year, the good fortune of being involved in experiments on this subject. I intend to report on this work. I would first like to offer my deepest thanks to Director Mitsuda for his kind guidance and the materials he provided.

Number of Cases.— The number of leprosy patients accommodated at our hospital fluctuates slightly each month due to factors such as death, escape, and transfer to other asylums. However, the average number of nodular leprosy patients over the course of one year, as surveyed by me, was 207. Of these, 64 patients, or 30.9%, suffered from erythema nodosum leprosum. Therefore, it can be said that approximately one-quarter to one-third of the nodular leprosy cases at our hospital were affected by this condition, making it a significant and common manifestation of nodular leprosy.

Relation to Sex.— Several more years of research would help to establish a more accurate picture of this relationship. Nevertheless, of the 64 cases, 48 were men and 16 were women, meaning that the number of women affected was approximately one-third of the number of men. This roughly corresponds to the male-to-female ratio within nodular leprosy cases. Whilst this may suggest a possible relationship [continuing into the future] between gender and leprosy, it contradicts the fact that (non-leprous) erythema nodosum is more common amongst women.

Relation to the Types of Nodular Leprosy.— When leprous granulomas form in the skin, they can be classified, as by Leloir, into two types: the subcutaneous and the dermal. Babes distinguishes three types: smooth infiltrations, miliary nodules, and elevated nodules. Unna also distinguishes three types, as has been previously mentioned. Although there may be more complex stages depending on the individual, location, and stage of the disease—which frustrate easy classification—for now, I shall broadly define two categories of nodules: lepromata forming in dome shapes on the surface of the skin—which we commonly refer to as the nodular type—and lepromata that are generalised, diffuse and irregularly bordered, which we simply classify as the infiltrative type. Lepromata that are buried under the skin, discerned by touch, and not elevated above the surface of the skin shall be provisionally classified as infiltrative. In what form of nodular leprosy did this condition more frequently occur? Our observations show that: 40 [of the patients suffering from erythema nodosum leprosum] were of what I have called the infiltrative type and 24 were of what has been defined as the nodular type. Therefore, this condition was more prevalent within the infiltrative type of nodular leprosy, accounting for the majority of cases, signifying that there is, to some extent, a disparity between the number of affected patients within each type. Moreover, there are also cases in which the administration of a medication such as chaulmoogra oil causes a previously nodular case to become infiltrative. In such cases, these patients, too, may experience an attack of this condition.

Relation to the Clinical Course of Nodular Leprosy.— Erythema nodosum leprosum typically occurs 5 to 12 years after the appearance of the early symptoms of leprosy. A particularly large number of patients experience it around the sixth year. Such patients accounted for 10 of the 64 cases. There were 3 cases in which the condition appeared 3 years after the onset of initial symptoms, and 2 cases in which it appeared after 28 years. In general, I was able to make a diagnosis of nodular leprosy in the fourth year following the onset of initial symptoms. Erythema nodosum leprosum may also develop within the fourth year. However, the majority of cases emerge at some point between the fourth year and the twelfth. It was within this period that the condition developed in one-fifth of nodular leprosy cases, and in more than half of all cases of [ENL]. It is

therefore considered that, rather than occurring in the early stages of nodular leprosy, eruptions [of ENL] tend to emerge several years into the course [of leprosy], with attacks remaining possible up to 28 years after initial onset. If this presumption is correct, it would imply that erythema nodosum leprosum can strike at any time during the life of a patient with nodular leprosy.

Age.— non-leprous erythema nodosum is more common during youth, so it is probable that erythema nodosum leprosum is likewise correlated with age. As seen in the attached table, it [ENL] is especially common during adolescence. In one regard, this fact corresponds well with the earlier discussion surrounding erythema nodosum leprosum and its occurrence 5 to 6 years after the initial manifestation of leprosy symptoms. In another regard, this helps to prove that, in the majority of cases of nodular leprosy, nodules do not appear at the onset but characteristically emerge after a certain period of time has elapsed. In the majority of cases in which initial symptoms of leprosy appear during puberty, eruptions of erythema nodosum leprosum tend to occur 5 to 6 years later. Such eruptions are peculiar to nodular leprosy and are, in the majority of cases, caused by erythema nodosum leprosum.

Clinical Manifestations[8]

The signs commonly observed during the course of our research shall be henceforth recorded.

1) General Symptoms

[Symptoms are] unclear in the premonitory[9] stage and may involve generalised or localised pruritus, hypersensitivity, neuralgia, arthralgia, headache, loss of appetite, general malaise, heart palpitations, and burning sensations. Symptoms vary and typically last between three and seven days. The typical symptoms of the condition are repeated rigors and chills accompanied by fever (39° up to and exceeding 40° [Centigrade]), headache, polydipsia, anxiety about the praecordia, and peripheral neuralgia. Eruptions appear a day later or sometimes within the same day. Eruptions first appear on the face before spreading to the flexor surfaces of the limbs. They are quite noticeable,

---

[8] Literally: Clinical Symptoms. The term 症状 has been used throughout in the original and can be literally translated as either *symptom* or *condition*. As this article was published before the application of medical interlingua, it is clear that, when translated into English, this term takes on a wider variety of meanings. When modified by an adjective such as “clinical,” a clearer translation would be “clinical manifestations.” I have generally translated it consistently as “symptom,” apart from in contexts in which this would not make sense.

[9] “Premonitory” chosen over “prodromal” in keeping with usage of the time.

scarlet in colour, range in size from the tip of the little finger to a two *sen* copper coin, and are both circinate and dome-shaped. Some eruptions may be solitary and some become confluent and generalised. Neuralgia as a result of nerve thickening, particularly in the median and ulnar nerves, is reported as being of an excruciating quality. Concomitantly, large joints may present with symptoms resembling rheumatoid arthritis, exhibiting articular hypersensitivity. The patient will suffer extreme agony. Eruptions are intensely painful in response to pressure, causing severe sleep disturbance often leading to exhaustion. Oedema occurs in the extremities as tension-type pain increases. Appetite is severely diminished, often leading to weight loss and emaciation. Protein can be found in the urine. The eruptive stage typically lasts two to three months before transitioning into a stage in which eruptions disappear. As eruptions resolve, fever abates and general symptoms gradually ameliorate. The skin may retain spots of blue pigmentation, or may be the seat of pruritus as the epidermis desquamates. At this stage, either during the stages of eruption or resolution, eruptions may soften as they are spontaneously absorbed, or they may suppurate, forming honeycomb-like ulcers. This may then be followed by a return to general symptoms as the skin becomes dry and creased and the patient significantly and systemically malnourished, in some cases eventually leading to death. However, in some fortunate cases, nutrition may improve and the patient recovers.

2) Respective Symptoms

Fever.— Body temperature elevates acutely to 39° and above, gradually becoming high-grade; becomes continuous during eruptive episodes and is alleviated as eruptions disappear. Patients may also become febrile during the recurrence or softening of eruptions.

Pulse.— Pulse rate increases apace with fever, reaching or exceeding 120 beats per minute, becoming rapid and tense. However, during bouts of prolonged and persistent fever, the pulse may become weak and irregular. This causes concern and anxiety to many patients.

Relation between Eruptions and the Seasons.— As previously mentioned, Director Mitsuda stated that eruptions often occur during periods of greater seasonal change, particularly in spring and autumn. In an attempt to observe the occurrence of eruptions in relation to the months of the year, it was found that they may occur in any month. However, there is a slight increase in incidence during seasons of greater climatic change, especially in the months of March and August when the transitions into spring or autumn occur. This is clearly supported by factual evidence.

As there is a certain relationship between the occurrence of eruptions and the seasons, so os there between eruptions and the weather on a given day. Eruptions tend to occur more frequently on cloudy or rainy days rather than on fine days. Hence, eruptions frequently occur when the weather is unsettled. Furthermore, there are often cases in which eruptions, which appear as small bumps on one day when the weather is overcast or rainy, suddenly disappear with the arrival of fine weather, causing a surprising shift.

Eruptions.—Eruptions vary greatly in size, ranging from the size of a lentil or the tip of the little finger to a two *sen* copper coin. They often appear disseminated across the body. A few lesions may appear on the face while a few may also appear on the limbs. In some cases, eruptions may be disseminated in a narrower sense, appearing localised to an area such as the face, while in others they may spread diffusely and concurrently over the body. In some cases, several lesions may become confluent, forming an indurated mass, spreading out in a diffuse manner, reaching the size of an egg to the palm of the hand. These eruptions are initially bright red in colour, situated deep within the dermis, and present as hyperaemic lesions, the surfaces of which are smooth, dome-shaped, and hemispherical, with well-defined borders. They are solid and somewhat elastic in consistency, and cause intense pain in response to pressure. This spontaneous nervous pain is so severe that when eruptions appear on the neck or back of the head, patients are unable to lie down, necessitating sleeping in a sitting position. In places such as the forehead, fingers, toes, dorsa and soles of the feet, pain is more intense owing to the fact that these regions have minimal subcutaneous tissue, instead being covered by a thick epidermal layer. The movement of the fingers and toes is impaired and there is a marked reduction in the grasping power of the hand. Additionally, due to hypersensitivity in the soles of the feet, walking becomes difficult. It is also reported that contact with clothing gives rise to immediate stabs of pain. Smaller joints become oedematous and their movement limited. Assuming a glossy appearance, [the skin] becomes pallid. Lesions gradually become more prominent, hands become rigid and 'claw-like,' the feet become enlarged and the margins of the soles become smooth. At this time, no pus can be seen if lesions are incised, only blood. The acute nature of eruptions is characteristic [of this condition]. New eruptions may occur next to existing ones, so that they coalesce. This, too, is characteristic, not being observed in cases of ordinary erythema nodosum. The [eruptive] lesions generally exhibit signs of regression after two weeks. The clinical signs [patterns] of regression vary [and can be characterised in the following ways]. 1) Hyperaemic [erythematous] patches may become bluish-brown in colour as they disappear. In some cases, this leads to a return to normal skin colour, with the hard nodules remaining palpable within the subcutis. These are considered signs of "pseudo-regression," in which a further attack may occur within a few days, bringing about a repetition of

symptoms. It is observed that patients may also experience symptoms as a result of the weather, especially cloudy weather. 2) The affected area shrinks in size. This pattern of regression is considered benign. This [regression] is accompanied by a pruritic sensation [pruritus]. The colour of the affected skin gradually changes from dark red to bluish-green, then to bluish-brown. The area then softens, loses elasticity, and is no longer tender. Although the area remains indented when pressed, it is not painful when pinched. Eventually, the affected area becomes dry, forming slightly hardened, fine wrinkles. The area may long retain a dark bluish-brown discolouration. This is particularly true for areas where lesions have coalesced or in areas of more severe past leprotic nodular activity. Conversely, there are also instances in which the affected area resolves completely, leaving no pigmentation or traces behind. 3) The affected area may present with changes in pigmentation similar to those described in the aforementioned pattern, but accompanied by severe epidermal dryness, [possibly] resulting in epidermal desquamation. Such desquamation appears pityroid and is particularly noticeable around the synovial joints, though at times it may appear membranous or lamellar. Once desquamation is complete, the skin becomes hard and stiff, forming fine wrinkles. 4) Lesions soften. This occurs a few days after the appearance of lesions, and is sometimes triggered by a slight rise in temperature. In some cases, softening occurs after a certain period of time, and is accompanied by further febrile episodes; whereas, in others, softening may occur in the absence of fever. This may also arise in patients exhibiting significant constitutional debility. When lesions soften, they do so gradually, either in an orderly manner, in a blossoming manner, or intermittently. The softened lesions, which had been [previously] elevated above the surface of the skin, become sunken or flattened. The affected skin attains a dirty, dark bluish-brown pigmentation without lustre. Occasionally, pustules may appear [on the surface], although softening may be overlooked [in such cases/presentations] owing to [the skin] often remaining intact. Lesions retain an indentation when pressed, are clearly demarcated from the surrounding skin, and present a texture akin to soft rubber. There is no tenderness or spontaneous pain associated with these lesions. The sizes of the lesions correspond to the [severity] of the infection. If a densely clustered area of lesions softens, the skin in that area becomes generally fragile and prone to breakdown in response to external stimuli. When the softened lesion is incised, its contents do not flow out rapidly but can be gradually expressed if pressure [is applied]. A highly viscous, dark-yellow mass may be discharged or, in some cases, a thinner purulent fluid. In cases characterised by the [former], a hemispherical cavity may be formed following the discharge of contents, leading to a natural [healing]. In some cases, however, honeycomb-like ulcers may form where multiple lesions have coalesced. Such cases tend to result in the formation of irregularly shaped, dirty ulcers. This may be associated with mixed infection. When the viscous contents are smeared onto a slide, dried, and subjected to lepra bacilli staining techniques—such as Gram's staining method or those simple

staining methods using aniline dyes—microscopic examination reveals the presence of leprosy bacilli, pus cocci, fibrous substances, nuclear debris, amongst other components. Leprosy bacilli often appear granular, irregular in length, and deeply-stained. Bacilli are found both inside and outside pus cocci, either scattered or in formations of globi. No other bacteria are observed through the application of other staining methods. Attempts to culture samples on agar or glycerin agar always result in no colony formation, yielding negative results.

When cases involving pustules or thin purulent discharge were examined in the manner previously described, the following were found: leprosy bacilli, pus cocci, nuclear debris, streptococci, Staphylococcus aureus, and diplococci, amongst others. These findings have been confirmed through comparison with results obtained from cultures.

In cases of softened lesions, observations have shown that lesions may be spontaneously [re]absorbed in the absence of suppuration. However, abscesses may form in some lesions as a result of [a] mixed infection, potentially being caused by external stimuli. [These abscesses] result in epidermal damage, leading to the formation of perforated, cavitary abscesses with uneven, dirty surfaces. In cases of coalesced or densely clustered eruptions, the softening and collapse of lesions may result in the formation of honeycomb-like ulcers. Of the 64 cases, 10 exhibited softened eruptions.

Each eruption shows signs of regression within approximately two weeks. However, eruptions may continue to appear, recur, soften, or form abscesses, resulting in irregular symptoms which may persist intermittently for two to three months or, in some cases, even longer.

Location of Eruptions.— Non-leprous or, as it is widely known, “common” erythema nodosum frequently occurs on the lower limbs. The lower limbs, and particularly the skin of the anterior surface of the tibia, tend to be the sites first affected. It rarely appears on the trunk or face. Conversely, in erythema nodosum leprosum, it is the face that is earliest and most often affected. The site of initial onset was the face in 33 cases; in 12 the thighs; in 6 the forearms; in 5 the lower legs; in 5 the head; and in 2 the upper arms. Within the face, it primarily occurs on the cheeks and mandibular area, while on the limbs, it tends to appear on extensor surfaces. However, it can also occur on the flexor and medial surfaces of the limbs, which is uncommon in the case of leprous nodules. Eruptions initially appear confined to a localised area but may subsequently develop in several other locations, accompanied by fever. In some cases, earlier eruptions subside as new ones arise, appearing to "chase" one another. The appearance and resolution of eruptions may also occur intermittently, as eruptions spread rampantly to various parts of the body as the disease reaches its acme. Affected regions may include the cervical, nuchal, thoracic, scapular, dorsal, abdominal, gluteal, penile, scrotal, palmar, manual, digital, femoral, Scarpa’s triangle, sural, dorsal foot,

plantar, and epigastric regions. Leprous nodules also tend to occur in these locations. It is therefore believed that this is why [eruptions of ENL] also develop in these areas. Although articular sockets are not affected, a few nodules occasionally form near the cubital fossa. Director Mitsuda has noted that [the distribution of eruptions] tends to correspond to the distribution of veins. My own observations frequently confirm this to be true, with eruptions more numerous on the extensor surfaces of the limbs. This is particularly apparent in emaciated patients where veins are easily visible on the flexor surfaces of the limbs. Eruptions often align with the branches of the median cubital vein or cephalic vein in the forearms, and with the internal and external saphenous veins in the legs, sometimes even appearing to penetrate the veins. Furthermore, in cases of leprous alopecia, eruptions tend to appear in areas of baldness whilst only sparingly in regions with remaining hair, further supporting this observation.

Furthermore, although an examination was conducted to determine whether eruptions occur in mucous membranes, conclusive results were not obtained. Hyperaemia of the palpebral conjunctiva is likely a result of other complications. While nasal obstruction was observed, it remains unclear whether this was caused by eruptions or hyperaemia. Similarly, findings for the oral cavity, pharynx, and larynx were inconclusive, necessitating further research.

Constitutional[10] Symptoms

Neuralgia.— Neuralgia, being present in 33 of the 64 cases, was therefore experienced by a majority of patients. It typically manifests in the peripheral zones of the body, often occurring at night while patients are lying down, or after they have engaged in light exercise such as walking. The pain varies in nature, from the quality of an electric shock, to a rheumatoid burning sensation, resembling the neuralgic stages of tabes dorsalis (or "pain tabes" as it is known). As eruptions spread throughout the body, neuralgia becomes more intense, persistent, and debilitating, hindering movement in the affected limbs. Such neuralgia is attributed to the thickening of nerve trunks and is predominantly caused by the thickening of the ulnar, median, and peroneal nerves. Thickening of the ulnar nerve is palpable between the forearm and upper arm, particularly in the upper part of the ulnar nerve groove, where it appears spindle-shaped or clavate, sometimes as thick as a little finger, and can be traced up to the axillary cavity. Thickening of the median nerve can be found between the upper part of the cubital region to the lower wrist flexor region, appearing spindle-shaped, with one particular case showing pronounced protrusion visible on the surface of the skin (refer to Fig. 5). Thickening of the peroneal nerve is often more pronounced, showing further increased

[10] Chosen over "systemic" to align with usage of the time.

thickening in some cases. Unlike the other two nerves, it does not exhibit specific peculiarities. All become tough and hard [on palpation], and patients report excruciating pain in response to touch. In two cases, thickened nerves had a soft texture, resembling that of a muscle upon palpation, though no beaded thickening was observed. Of the 64 cases, ulnar nerve thickening was notable in 29; median nerve thickening in 25; whereas peroneal nerve thickening could not be conclusively quantified. Additionally, thickening of the great auricular and posterior cervical nerves was observed but was not prominent.

Arthralgia.— Initially being observed in the major joints of the extremities, arthralgia becomes more pronounced in joints neighbouring the largest number of eruptions as the outbreak progresses, often presenting a migratory pattern, shifting intermittently. The extremities of joints may become enlarged and thickened, displaying pronounced signs of synovitis. This condition is particularly evident in the first and second finger joints, frequently affecting the index and little fingers and the thumb. While the severity of pain varies, even in moderate cases, both passive and active movements may elicit severe pain. Consequently, patients can only manage minimal joint flexion and exhibit hypersensitivity in response to pressure. Other finger joints are particularly distinctive, showing similarities to acute articular rheumatism [rheumatoid arthritis]. They exhibit thickening and swelling, with the skin over the joint losing its folds, appearing pallid, smooth, and leaving slight indentations upon pressure. Among all joints, the knee is most commonly affected, followed by the ankle, shoulder, elbow, and wrist joints. However, joint pain caused by these eruptions generally [affects] multiple joints; it is rarely isolated. Of 64 cases, 32 reported joint pain, constituting half of the cases. Of these, pain was first felt in the knee joint in 7 cases; in the ankle joint in 5 cases; and in the wrist joint in 3 cases.

Mild cases.— Even in mild cases, arthralgia may be felt during activities such as walking or bending and stretching. Additionally, at night when the patient is at rest or lying down to sleep, a burning sensation in the affected side may precede the onset of pain. This pain is often described as a burning sensation.

Respiratory System.— If leprous nodules form in the throat or larynx, patients may complain of difficulty breathing, accompanied by fever and intense thirst. Breathing may become shallow and rapid, though not severely so. Two patients who had previously suffered from pleurisy experienced a recurrence [of pleurisy] due to this condition [ENL]. No other abnormalities were noted.

Circulatory System.— It is not uncommon to observe an irregularity in the heart's apex beat, with an accentuation of the first heart sound. Details regarding the pulse have been mentioned earlier.

Digestive System.— The tongue may become dry and coated with a thick layer of fur, occasionally appearing sooty. Cracks can form on the lips. Patients often complain of gastric hypersensitivity, lack of appetite, and gastric stasis. Diarrhoea may also occur.

Spleen, Liver, Lymph Nodes, and Testes.— In nodular leprosy, the spleen and lymph nodes typically exhibit oedema, though it is challenging to provide conclusive findings in practice. However, during thorough examinations, splenic enlargement can sometimes be observed, and patients may complain of pain in the splenic region. While lymph nodes are not notably enlarged, they may exhibit a slight increase in hardness during the acme of eruptions. This phenomenon is often attributed to mixed infections associated with the eruptions. As for liver enlargement, no clear observations have been made, and it is unlikely that any significant enlargement occurs. As the testes, like the skin, may develop nodules in cases of nodular leprosy, it is crucial that close attention be paid to any eruptions occurring in this area. During the acute phase of the disease, the testes are particularly sensitive to pressure, causing significant pain, especially in the tail of the epididymis. However, testicular swelling itself is not clearly apparent. Further experimental investigations and reports on this matter are anticipated.

Urinary Changes.— In nodular leprosy, chronic nephritis is frequently observed as a complication, though a detailed examination is deferred for later study. It is a fact that, with the onset of eruptions, patients often exhibit marked proteinuria and various types of casts. While it is necessary to differentiate this from febrile proteinuria, cases where facial oedema and proteinuria occur before eruptions, and where eruptions develop within two to three days after prescribing rest, suggest that nephritis may indeed be caused by the disease itself. Among the patients, nine individuals experienced nephritis induced by this condition without any prior history of nephritis.

Haematological Changes.— Based on the examination conducted by Director Mitsuda on May 29, Meiji 44 [1911], the blood analysis of a patient named Ozawa revealed the following: small lymphocytes 10.25%, large lymphocytes 3.88%, transitional forms 4.46%, eosinophils 0.58%, polymorphonuclear leukocytes 79.29%. The total white blood cell count was 15,700; and the red blood cell count was 4,237,500, with a ratio of approximately 269 red blood cells per white blood cell, indicating leukocytosis. Further detailed observations on blood analysis will be provided as supplementary notes at a later time.

Hair Loss.— During periods of active eruptions, hair loss on the scalp frequently occurs, and eyebrows are similarly affected. Once all eruptions have completely disappeared, hair will generally regrow. However, when softening, tissue damage, and ulceration occurs, the mechanism for regrowth is lost.

Mental Condition.— In general, the mental state remains clear, with no instances of confusion observed. However, insomnia may occur. During febrile episodes, patients may exhibit delirium.

Histological Findings

The samples used for histological examination were obtained from six cases of nodular leprosy, all classifying as the infiltrative type. Specimens for microscopic analysis were selected from eruptions that had occurred within the past few days. Each skin sample was fixed in formalin solution, hardened in alcohol, and converted into celloidin sections. For routine examination, haematoxylin, eosin, and Van Gieson's solution were used for counterstaining. For the staining of leprosy bacilli, the Ziehl[-Neelsen] or Guerbet method was employed. Alternatively, the samples were treated with Ziehl-Neelsen solution, decolourised using 25% sulphuric acid, and then counterstained for microscopic examination with Weigert's iron haematoxylin solution. In some cases, Van Gieson's solution was also used for staining to prepare specimens for further analysis. To investigate the presence of bacteria other than *Mycobacterium leprae*, methylene blue as a simple stain or Gram's staining method was used for comparison. For staining elastic fibres, Weigert's resorcin fuchsin solution was applied. Plasma cells and mast cells were stained using Unna's polychrome methylene blue and then decolourised with a glycerin ether mixture before being subjected to examination.

Case 1: Haneishi X[11]

Nodular leprosy, infiltrative type

Age: 21 years

Duration of illness: 9 years

[11] Patient names for all cases are presented as "[Family Name] Someone," with personal names not being divulged. I have replaced this with "X" in all cases.

Around March of the previous year, the patient developed erythema nodosum leprosum, appearing in various areas of the face. By April, peculiar rashes appeared on the extensor surfaces of the head, upper arms, forearms, and thighs. These were occasionally accompanied by ulnar nerve pain. However, the patient reported no chills or fever, aside from mild symptoms at the initial onset of the eruptions. Joint pain was absent, but once eruptions appeared, the patient experienced significant nervous pain and complained of hypersensitivity. A distinct, dome-shaped lesion described as a "typical eruption appearing on the fifth day post-onset" was observed on the ulnar side of the antebrachium. This was examined using the treatment methods outlined earlier. The findings are detailed below.

No significant changes were observed in the epidermal layer. In the dermis, leprous infiltration was mild, localised around hair follicles and sweat glands. These areas were infiltrated with morular vacuolated leprosy cells, most of which being mononuclear. The blood vessels were dilated, and haemorrhagic foci were found in the reticular layer, accompanied by haemosiderin granules. Vessel lumen were frequently found to be completely filled with lobulated or polynuclear leucocytes.

The main [pathological] changes were observed in the fatty tissue, which was clearly divided into areas of loosely packed and moderately dense cellular infiltration. The former [loosely packed] were primarily composed of [giant/clumped] polynuclear vacuolated leprosy cells with some epithelioid cells present. Many of these cells had large, [mononuclear-like 單核胞狀] nuclei; a few giant vacuolated/vacuole cells were also intermittently found. These areas also showed infiltration by a small number of polynuclear leucocytes. This was particularly marked in areas located near the infiltration foci of the latter [moderately dense]. Cellular infiltration of the latter consisted mainly of colonies of polynuclear and lobulated leucocytes, loosely intertwined with fine connective tissue fibres. Occasionally, mononuclear or polynuclear leprosy cells, epithelioid cells, and adipocytes were intermixed. All cells were atrophied, with [deformed 變形] nuclei. Blood vessels running through these areas contained abundant polynuclear leucocytes, showing significantly narrowed lumens due to the pressure [exerted on them].

Varying amounts of migrating leucocytes were observed in the connective tissue of the interlobular and deep reticular layers, although no significant accumulation was found. The connective tissues of these areas showed fluid exudation, causing the fibres to loosen and separate. Findings attained from stained bacilli samples showed that leprosy bacilli were found parasitising the vacuolated leprosy cells, primarily in the vacuole peripheries, appearing granular and irregularly arranged. Leprosy bacilli were also observed in small, regularly shaped globi as well as individually. However, at the centres of polynuclear leucocyte cluster foci, leprosy bacilli often

appeared poorly stained. Occasionally, free-floating leprosy cells were present; however, cells hosting scattered leprosy bacilli existed only in the surrounding areas. Our research reveals that polynuclear and lobulated leucocytes host leprosy bacilli, and these bacilli can also be found within the nuclei. Leprosy bacilli are also frequently present within connective tissue cells. These leprosy bacilli appear morphologically indeterminate as they develop into granular forms, although some retain a regular shape. Using Gram's staining method and single-staining with methylene blue, no other bacteria were identified.

Plasma cells were concentrated around the small arteries, with mast cells scattered throughout, including in polynuclear leucocyte clusters. We were unable to confirm the existence of leprosy bacilli within any of these cells. All veins exhibited endophlebitis and periphlebitis, providing a favourable environment for leprosy bacilli. Small veins consistently showed multi-nucleated leukocytes. The arterial walls were thickened. The arterial walls were thickened. While leprosy bacilli were not found within the endothelial cells of the arteries, they were occasionally observed in the outer membrane. No thrombotic occlusions were present in either the arteries or veins. Leprosy bacilli were frequently detected in both the neural substance and outer sheath. However, no changes were observed in the elastic fibres. (Examination Date: April 3, Meiji 44[12])

Case 2: Same Individual

In late July, the patient experienced a recurrence of symptoms. Scattered eruptions appeared on the face and the extensor surfaces of the upper and lower limbs. By August, the eruptions had increased significantly, and by mid-August, the patient was compelled to remain bedridden. At this time, the patient complained of swelling and pain in the knee joints, ankle joints, and wrist joints. Concurrently, thickening of the median and ulnar nerves was observed, accompanied by severe neuralgia that interfered with sleep. Febrile episodes repeatedly reached temperatures as high as 42° [Centigrade]. During this period, a third bout of eruptions appeared, covering almost the entire body, sparing only the anterior abdominal region. These eruptions fluctuated in presence and intensity without a consistent pattern, with the entire episode lasting over three months. On August 7, while the patient still exhibited mild fever, I collected a biopsy sample from a slightly dark reddish eruption that had developed on the ulnar side of the upper right forearm a few days earlier and prepared histological specimens.

---

[12] 1911

The infiltration of leprous tissue into the dermis was mild overall. A large number of connective tissue fibres were observed in which blood vessels were dilated and abounded with leucocytes, showing signs of inflammatory oedema.

Within the adipose tissue, a significant amount of polynuclear leucocyte colonies were found at the foci of lesions. Consequently, vacuolated leprosy cells showed marked atrophy, with irregularly shaped nuclei, appearing stellate or polygonal. Staining for leprosy bacilli was uneven, and isolated vacuolated cells devoid of leprosy bacilli were present. Epithelioid cells were also reduced in size, although individual leprosy bacilli were observed within some of these cells. Some polynuclear leucocytes exhibited phagocytising activity, while eosinophilic cells were also sporadically present.

Adipose cells were gradually compressed and separated, with leucocyte infiltration increasing between them, contributing to an expansion of leucocyte-dense lesion sites.

A band of connective tissue surrounding colony foci showed cellular proliferation, with some cells harbouring parasitic leprosy bacilli. Scattered vacuolated leprosy cells, leprosy globi, and epithelioid cells (some containing bacilli) were also observed. In addition, the lesions were invaded by polynuclear leucocytes, with leucocytes permeating the spaces between adipose cells. Veins in the affected areas were abundant with polynuclear leucocytes, with endothelial cells also being parasitised by the leprosy bacilli, although no leprosy-related emboli were observed.

Case 3: Mashimo[13] X

Nodular leprosy, infiltrative type

Age: 30 years

Duration of illness: 17 years

Since June of last year, the patient has suffered from persistent erythema nodosum, with eruptions appearing and disappearing irregularly. Subjective symptoms were mild. In September of last year, a dark-reddish eruption approximately the size of the tip of a little finger was observed on the central part of the extensor surface of the right thigh.

The focus of the leprous infiltration was located in the upper/superficial layer of the dermis and consisted of fine, vacuolated leprosy cells. Infiltrations of large vacuolated leprosy cells were

---

[13] Japanese surnames, always written in kanji (Chinese characters) can usually be read in a variety of ways, with several readings usually existing for the same characters. Accurate readings can often only be gained if the person to whom the surname belongs reveals how it is pronounced. So, here, although in most cases this surname (真下) is read as “Mashimo,” it may also be read as “Mashita.”

found in the adipose tissue. The interlobular connective tissue also showed an abundance of leprosy cells.

Clusters/colonies of polynuclear leucocytes were moderately pronounced, occupying two to three lobules of the adipose tissue, forming colonies that were interspersed with areas containing milder polynuclear leucocyte infiltration. The central cluster/colony was the largest, with a central area displaying degenerative leucocytes and their fragments. However, the peripheral clusters/colonies, as well as other [colonies], showed leucocytes densely aggregated, interspersed with polynuclear leprosy cells and epithelioid cells, which were sometimes parasitised by leprosy bacilli. Most of these cells exhibited atrophic changes.

The blood vessels within the clusters had narrowed lumens, often filled with polynuclear leucocytes. Small globi of leprosy cells were scattered about the periphery of smaller clusters. Eosinophilic cells were observed with minimal leucocyte infiltration in the foci of leprous infiltrations and interlobular connective tissue.

In the small-sized veins, occlusive phlebitis was noted, evidence by the notable presence of leprosy bacilli across the layers. These veins contained slightly more leukocytes than erythrocytes. The arteries were thickened, but no bacterial emboli were observed.

At the site adjacent to the fascia, there was an accumulation of small round cells with only a small number of free-floating leprosy bacilli present.

Case 4: Ota X

Nodular leprosy, infiltrative type

Age: 31 years

Duration of illness: 5 years

The patient first experienced [eruptions of] netsukobu[14] this year, and has since experienced three bouts. A specimen was prepared at the time of the third bout of attacks. Initially, a bright red lesion, the size of a two-sen copper coin, was observed on the mid-region of the anterior thigh. The lesion was hard, exhibited tenderness to pressure, and caused spontaneous pain. Subsequently, similar eruptions appeared on the head, face, lower limbs (anterior surfaces), scapular region, nape, and neck. The patient experienced several episodes of chills and rigors, along with ulnar nerve pain and plantar neuralgia. The ulnar nerve exhibited significant thickening at the upper part of the cubital groove. A lesion on the left forearm, at the radial styloid process, was excised on the second day of its appearance and submitted for examination.

14

Pigment in basal cells was found to be very minimal. Connective tissue was abundant in the dermis. Aside from the periphery of hair follicles, leprous infiltration was generally minimal. The cells were primarily mononuclear, with notable lymphocyte admixture. Vacuoles and globi were rarely formed. Proliferation of connective tissue cells was observed, with many blood vessels dilated, giving the overall appearance of a fibroma.

In contrast, the fatty tissues showed a high degree of leprous infiltration. The cells therein located were primarily vacuolated mononuclear or polynuclear cells. Small globi were often observed. Epithelioid cells were likewise predominantly vacuolated,[15] mononuclear and host to leprosy bacilli.

Polynuclear leucocytes were prominently present across all layers, penetrating up into the intercellular spaces between the granular cells of the epidermis, whilst also gradually infiltrating deeper layers. As they progress from the dermis to the fatty tissues, they accumulate to form clusters, compressing and causing the atrophy of vacuoles and adipocytes. These clusters coexist with epithelioid cells, though only in small quantities. The presence and parasitism of leprosy bacilli are minimal, and phagocytic leucocytes containing bacilli are sporadically observed across all layers. The veins, compared to the arteries, tend to contain greater numbers of leucocytes. Leprosy bacilli are consistently found within the endothelia and tunicae externae of veins. There is also a notable presence of leprosy bacilli in nerve fibre fascicles. Furthermore, eosinophilic cells are markedly abundant in the interlobular connective tissue and in the periphery of leucocyte clusters.

Case 5: Takahashi X

Nodular Leprosy, Infiltrative Type

Age: 60 years

Duration of Illness: 19 years

The skin on the face and the extensor surfaces of the limbs was markedly discoloured, presenting with diffuse infiltrations of a copper-brown hue characteristic of nodular leprosy. Eruptions first appeared at the beginning of last September, on the anterior jaw, followed within several days by pea to broad-bean-sized dome-shaped eruptions on the extensor surfaces of the upper and lower limbs and the neck. These eruptions caused the affected areas to appear a dark blackish-copper colour. The patient complained of neuralgic pain in the lower legs but reported no arthralgic pain. The patient experienced fever, headache, and intense thirst. Examination was

[15] Abbreviation used in the original (literally: *cell-form* mononuclear epitheloid cells) so that this may be interpreted in several ways. However, considering the context and the grammatical use of "likewise," it can be assumed that this is referring to "vacuolated mononuclear epithelial cells" with "cell" here being a shorthand for "vacuole" (which, in Japanese, is composed of the characters meaning "empty" and "cell").

conducted on a dark red eruptive lesion with minimal pigmentation located on the skin at the side of the neck.

The epithelial layer was flat, with the papillae having almost entirely disappeared. The granular cells were round and limited to one or two layers, while the basal cells were cuboidal and contained a conspicuously large amount of pigment.

The dermis exhibited a high degree of cellular infiltration, divided into sections by thin connective tissue, forming round, elongated, or oval shapes. These cells were primarily composed of fine, polynuclear vacuolated leprosy cells, with small globi also notably present. Blood vessels were dilated and filled with blood corpuscles. The endothelial cells were laden with parasitising leprosy bacilli.

Infiltration in the adipose lobules consisted mainly of large vacuolated leprosy cells. However, in some areas, these cells were eroded and occupied by polynuclear leucocytes, leading to atrophy of the leprosy cells.

Leucocytes, predominantly lobulated and polynuclear, strongly infiltrated the dermis and, in some areas of the fatty tissue, accumulated into clusters. Within the lobules, on the other hand, the infiltrative leucocyte foci formed. No leprosy bacilli were observed within their central areas. However, leprosy bacilli were present in the surrounding and interlobular connective tissue, with some also being found within connective tissue cells.

The arteries showed thickening, within which a small number of leprosy bacilli were observed.

The sweat glands and hair follicles were atrophied in areas of infiltration, but remnants were preserved. Leprosy bacilli and leucocytes were identified in the epithelial cells of the hair follicles.

Plasma cells were observed around the small blood vessels; leprosy bacilli were absent. Mast cells were scattered throughout, showing no abnormalities.

Leucocytes infiltrated the outer nerve sheath, where leprosy bacilli were also present. No other parasitic bacteria were detected, and there was no evidence of bacterial embolism.

Case 6: Ozawa X

Nodular Leprosy, Infiltrative Type

Age: 23 years

Duration: 10 years

In March of last year, the patient suffered from a severe case of nodular erythema, which resolved after four months. However, subsequently, the patient occasionally developed eruptions that, although afebrile, were painful. A lesion that penetrated a small branch of the right basilic vein was examined microscopically. The lesion was observed three days after its onset.

Leucocyte infiltration was localised to the fatty tissue, forming a moderate lesion with polynuclear cells. In the lower central part of the lesion, leucocytes showed mild pigmentation and nuclear fragmentation, along with chromatin granules. Surrounding this area, distinctive polynuclear leucocytes were found in dense clusters. Vacuolated cells containing granulated leprosy bacilli were intermixed with vacuolated cells that did not contain the bacilli. Epithelioid cells and mast cells were predominantly located around the periphery of the clustered lesions.

Infiltrative leprous lesions in the fatty tissues showed a higher degree of leucocyte activity compared to those found in typical cutaneous leprosy; this infiltration was even more pronounced than that of the leprous lesions in the dermis.

A small branch of the right basilic vein, classified as a moderately sized blood vessel, was located within the deeper fatty tissue without a direct relationship to the clustered lesion. A small number of leprosy bacilli were found within the endothelial cells of this vein. The vessel intima showed slight thickening, while the adventitia exhibited significant infiltration, containing a great number of leprosy bacilli. However, no leucocyte infiltration was observed around the adventitia. In contrast, smaller veins and capillaries, which appeared to be part of the same venous system, displayed slightly dilated lumina; these contained leucocytes. The surrounding areas of these vessels showed leucocyte infiltration.

Connective tissue cells showed a certain degree of proliferation as well as inflammatory oedema. In several places, phagocytosis of the leprosy bacilli by polynuclear leucocytes was observed.

Clinical Course.— This varies dramatically. In mild cases, regression may occur after 2 weeks; in moderate cases, complete disappearance and healing make require 2 to 5 months. In 21 of the 64 cases [the eruptive period] lasted for 2 months; in 3 cases it lasted for 3 months; in 4 cases it lasted 5 months; and in one case alone did it persist for a year. Hence, one can say that, on average, the complete resolution [of eruptions] requires 3 to 4 months.

Why is it that some patients experience a recurrence of this condition? In 7 of the 64 cases, patients experienced 3 further attacks within a year; in 5 cases, there were 4 recurrences within a year; and only in one case was there more than 5 further attacks within a year. In only one case did bouts of eruptions constantly appear and disappear throughout the course of the year. During eruptive periods, experienced only mild localised pain, the patient continued to engage calmly in their labour,[16] showing no other.

[16] It is unclear from the original whether this is metaphorical or literal. It may mean that they continued labouring (as a form of work) or that they continued to battle through the cruel laboriousness of their struggle with the condition.

Factors that must be considered when talking of [clinical course] are; the weather (especially times when its changes are greater); food and drink (such as animal fats and tree nut oils); and the individual's hygiene, passions, and other such factors, which may greatly influence [the course of the condition].

The appearance of eruptions, course [of the condition], and recurrences [of eruptions] also differ according to the constitution of the individual. As previously mentioned, this condition is more common amongst young leprosy patients and frequently affects those leprosy patients who maintain their health, ensuring a constant engagement in strenuous labour and physical activity. On the other hand, this condition also affects the debilitated, the anguished, and those who, suffering from other complications, have been plunged into pitiable conditions verging on death. Recurrences are similarly and inevitably varied, seeming to frequently occur in patients who are healthy but intemperate, as well as in patients who suffer from other complications. Among patients who developed this condition, the following complications were observed:

Tuberculosis in 4 cases;

Chronic nephritis in 7 cases;

Pleuropneumonia in 3 cases;

Tuberculous lymphadenitis in 3 cases, and;

Mitral incompetence in 1 case, amongst others.

Since Uffelman, Pfeiffer, and Oehme harboured some doubts about the relationship between (non-leprous) erythema nodosum and tuberculosis, I make this record merely so that it may be used as reference material.

Sequelae.— May include nephritis, nerve thickening and pain, trophic [17] ulcers, constitutional debility, alopecia. It is sometimes due to generalised weakness that patients' suffering terminates in death. This was seen in two cases. It is rare for even frequent eruptions directly cause death.

Differential Diagnosis.— It is particularly important to distinguish eruptions relating to this condition—as complications of nodular leprosy—from erysipelas. By no means is this task simple and it becomes even more challenging when eruptions [of ENL] have coalesced to form diffuse infiltrations. It is only clear which one is which when one subjects both to a thorough examination.

---

[17] Literally: "festering ulcers," it is clear that this refers to "trophic ulcers."

	Erysipelas	Erythema nodosum leprosum
1)	Affected areas appear dark red and oedematous	Affected areas appear bright red and elevated
2)	Diffuse infiltrations accompanied by a painful burning sensation	Localised nodular infiltrations with nervous pressure pain and hypersensitivity
3)	Bullae often form in affected areas	Bullae never form in affected areas
4)	As inflammation subsides, area becomes discoloured and gradually less oedematous	As inflammation subsides, changes to the pigmentation of the affected area becomes clear
5)	Proven by presence of erysipelas cocci	Proven by presence of rod-shaped leprosy bacillus
6)	Very rarely results in [tissue] softening or collapse	Often leads to [tissue] softening and collapse
7)	Painful swelling of all lymph nodes at the affected area	Is not characterised by peculiar swelling of the lymph nodes
8)	Aside from the face, migrating erysipelas affects the upper and lower limbs, although locations are often indeterminate	Aside from the face, mainly affects extensor aspects and surfaces of limbs
9)	Not accompanied by arthralgia, only by a feeling of heaviness in the joints	Accompanied by arthralgia resembling that of articular rheumatism [rheumatoid arthritis]
10)	Rarely involves neuralgia	Often involves neuralgia
11)	Approximate 3 week clinical course with severe constitutional symptoms	Symptoms usually begin to fade after 3 months; generally leads to a comparatively less severe condition

Next, a condition with which [erythema nodosum leprosum] must be distinguished is the non-leprous variety of erythema nodosum. Although the latter seldom occurs in leprosy, these two conditions must naturally be differentiated. The main ways in which this can be done are by remembering that:

1) In both conditions, the locations of occurrence are different;
2) In erythema nodosum, lesions do not coalesce or merge;

3) In erythema nodosum, eruptions very rarely appear on the face;

4) In erythema nodosum, lesions do not soften;

5) In erythema nodosum, the clinical course is comparatively short;

6) In erythema nodosum, females are affected more often than males;

7) Erythema nodosum leprosum is histologically peculiar in that inflammation is primarily formed of infiltrative clusters of polynuclear leucocytes within leprous granulomata found within subcutaneous fatty tissues;

8) Although arthralgia may be a feature of erythema nodosum, neuralgia is not.

Other acute facial swellings associated with leprosy may resemble confluent [patches of] erythema nodosum leprosum in terms of diagnosis. However, when eruptions occur on other parts of the body, remembering that this condition is specific to nodular leprosy makes it easy to distinguish between the two.

However, whether eruptions occur on other parts of the body or not, keeping in mind that this condition is specific to nodular leprosy allows for a clear distinction to be made. Dr Sugai's[18] paper, on the histological differences in distinguishing nodule softening unrelated to pyogenic bacteria, addresses this differentiation.

Prognosis.— Judgements of prognosis must be made with prudence, as the prognosis depends on the presence of complications. Among the 64 patients, 3 died. One of those who died had a coinfection of pulmonary tuberculosis; another patient suffered from concomitant beriberi. The third died as a result of septicaemia. Hence, it has not been possible to arrive at a mortality rate directly relating to this condition. In other words, fatalities linked to this condition tend to be caused either by severe complications which overwhelm the body to the extent that eruptions may appear, and because of which death is caused; or, due to the preexisting complications themselves. This much is doubtless. However, there are some cases in which this the eruptions of this condition appear at a time of concomitant complication-related suffering, thereby leading to much poorer prognosis. Although the prognosis of each individual eruption may be good, if eruptions appear almost entirely cover the body, the patient will certainly experience severe constitutional symptoms, which give rise to a gradual and grave worsening in condition. And yet, at least in comparison with the severity of the unexpected constitutional symptoms experienced, the prognosis may still be good.

---

[18] Takekichi Sugai, Japanese dermatologist and Hansenologist, 1871–1944

The prognosis is not good in cases where lesions soften, become damaged, and, due to mixed infection, thereby ulcerate. We observed one case in which a patient developed sepsis in this manner, and died.

Cases of isolated lesion softening not caused by mixed infection may lead cause patients to develop dangerously high fevers. However, if prudence is upheld in the examination room, such lesions can be identified, then promptly excised, drained of their pus, and the patient saved.

It is for this reason that it feels justified to claim that, although this disease may lead, in some cases, to systemic exhaustion and weakness, the prognosis is typically fair.

There is no significant relationship between the factors of sex and age, and prognosis. Pulmonary disease, kidney disease, beriberi, anaemia, poor health, heart disease. All these complicating factors are far more important when it comes to prognosis.

Broadly speaking, the greatest dangers associated with this condition are: 1) the substances of inflammation and the ways in which they infiltrate the heart and kidneys; 2) history of complicating conditions; 3) the constitutional predisposition as well as the peculiar attributes of the individual.

Treatment

Prevention.— Since the causative agent of this condition remains unidentified, specific preventive measures are unclear. However, considering that it tends to occur during seasonal transitions in spring and autumn and often affects individuals with poor hygiene and unhealthy habits, attention should be given to these factors in patients. In particular, patients who are already suffering from other complications should exercise caution.

Observations and Hypotheses.— Histological findings and blood tests indicating an increase in white blood cells suggest the presence of phagocytic activity as described by Metchnikoff. It has been observed that leprosy bacilli and their associated nodules diminish or disappear, leading to the hypothesis that methods to provoke or stimulate this phenomenon could theoretically serve as a basis for serotherapy. After observing patients who have recovered from this condition, the nodules fade, leaving behind wrinkling and pigmentation. The transformation from the previous leonine facial appearance to an aged and softened facial contour (broad, relaxed, with fine and coarse wrinkles) is notable. Thus, in cases without fever or severe systemic symptoms, this treatment approach may be regarded as highly effective against lepromata.

Drug Therapy.— From a practical standpoint, reducing the frequency of outbreak occurrences, regardless of its theoretical importance, is significant because severe systemic

symptoms resulting from eruptions cause great suffering to patients. Although no definitive cure exists for this disease, symptomatic treatments remain the primary option.

Nursing and Diet.— Patients with fever should be confined to bed, ensuring physical and mental rest. Ice pillows and cold packs should be applied to the head, and the patient's position should be regularly changed to prevent bedsores, which must not be overlooked. Given the febrile nature of this disease, dietary protein supplementation is necessary to counteract protein breakdown. Adequate nutritional support should be provided. Commonly, milk is administered, which has proven effective in preventing or treating nephritis frequently associated with or recurring during this disease. This approach is considered suitable for its therapeutic, preventive, and nutritional benefits. Other dietary considerations should follow general guidelines for febrile illnesses. During febrile periods, hydrochloric acid quinine is administered for antipyretic purposes, while cold water or hydrochloric acid lemonade is provided to alleviate thirst. To invigorate the heart and overall vitality, small amounts of red wine may be added.

Inductive Therapy.— Laxatives have been employed for their effectiveness. Recently, oral administration of limewater has shown promising results. For neuralgia and joint pain, drugs such as sodium salicylate, aspirin, phenacetin, lactophenin, and antipyrine have been used, with sodium salicylate showing the most efficacy. Dr. Mitsuda has injected "tetrodotoxin," which has proven effective not only in alleviating symptoms but also in halting eruptions.

Management of Eruptions.— For eruptions, a 10% ichthyol ointment is generally applied to the affected area, along with the "Blow solution" compress. In five cases, I performed shallow incisions on the eruptions and treated them with lime-acid warm compresses, achieving remission. When softening of eruptions occurs, early application of sterilizing and disinfecting measures is crucial. Incisions are made to drain pus, followed by bandaging with lime-acid compresses. This not only prevents dangerous secondary infections but also addresses ulcers caused by spontaneous breakdowns, which are notoriously difficult to heal. During secondary infections, streptococcus may be identified through microscopic examination and culture. In such cases, anti-streptococcal serum injections have expedited recovery. Additionally, maintaining oral hygiene and facilitating proper excretion of waste are essential. Appropriate medications and physical therapies should be employed for complications as needed.

I shall now provide a summary key points, as contained within the paper above:

1) Erythema nodosum leprosum may only occur in cases with nodular leprosy, in one-third to one-fourth of all such cases.
2) Eruptions are more often infiltrative than nodular, in a ratio of 2 to 1.
3) Sex incidence of erythema nodosum leprosum follows that of nodular leprosy.
4) Eruptions of erythema nodosum leprosum are not initial events, usually appearing about 6 years after the discovery of initial symptoms.
5) Erythema nodosum leprosum occurs mostly in adolescence, something which provides strong evidence for the fact that, when the initial symptoms of leprosy appear during the onset of puberty, the disease often progresses to the nodular leprosy during [later] adolescence.
6) The clinical features of erythema nodosum leprosum include those typical of common eruptive febrile diseases.

I. The latent period is inconstant and mostly uncertain.

II. Eruptions appear with attacks of fever accompanied by rigors and chills, although afebrile occurrences of erythema nodosum leprosum may also occur [afebrile erythema nodosum leprosum].

III. Eruptions range from the size of a lentil to a two *sen* copper coin, are bright red in colour, solid and elastic in consistence, elevated, and hypersensitive. Eruptions may be solitary or confluent (confluent erythema nodosum leprosum/localised erythema nodosum leprosum). Eruptions tend to first appear on the face, but later in those regions where lepromata occur, as well as on the flexor surfaces of the limbs where lepromata do not usually occur. The distribution of eruptions corresponds to that of the veins.

IV. Erythema nodosum leprosum occurs chiefly in the spring and autumn, especially at time when the seasons change.

V. Colour changes during the disappearance of eruptions are from dark red, to bluish green, then bluish brown.

VI. After eruptions have disappeared, residual findings may be of: (a) dryness and creases of the skin with bluish pigmentation; (b) there being no traces of eruptions left at all; (c) desquamation of the epidermis or; (d) softening of the skin.

VII. Softening of eruptions during their clinical course may (or may not) be associated. with the detection of suppurative germs, in which case dirty, irregularly-shaped ulcers containing

[19] A lot of this summary was cross-referenced or quoted from: http://leprev.ilsl.br/pdfs/1958/v29n2/pdf/v29n2a07.pdf, in which an earlier translation of the summary was provided. This summary does not include all points mentioned in the original. These have now been added.

yellow liquid pus may be formed. In the absence of suppuration, a thick glutinous orange-coloured clot of pus may be discharged. This varies.

VIII. Neuralgia as a result of nerve enlargement is prominent amongst the constitutional symptoms. Arthralgia as a result of synovitis is next in prominence. There seem to be no marked changes in the urine, nor in the internal organs.

IX. [Pathological] changes occur in the blood, causing leucocytosis.

7) The clinical course may be acute, subacute, or chronic. It is most often acute. Hence, I would define the following clinical courses:

(1) (Common) acute erythema nodosum leprosum

(2) Protracted erythema nodosum leprosum

(3) Long-term erythema nodosum leprosum

9) The essential pathological structure is of inflammatory formations within fatty tissues, and of infiltrating colonies of polynuclear leucocytes.

10) In diagnosis, it is important to differentiate [this condition] from erysipelas, which may accompany nodular leprosy.

11) Prognosis is generally benign.

12) At present, there is no specific remedy for erythema nodosum leprosum, although calcium preparations, quinine hydrochloride, and laxatives demonstrate favourable action.

Finally, I would recommend the [clinical] designation of erythema nodosum leprosum. Not only does this condition clinically resemble erythema nodosum, and is a characteristic syndrome appearing in the course of leprosy (especially nodular leprosy), but also due to the need to distinguish this condition from the "erythema nodosum" (a term which has already been approved as a clinical entity) caused by Dr Shitachi's potassium iodide. The histological differences found within this condition moreover support the need for its designation. The term erythema nodosum leprosum is, therefore, a term which reasonably may be adopted for this singular and independent clinical syndrome.

Cases

(1) *Nakamura X*[20]
Nodular leprosy, infiltrative type
38 years of age
Male
(Meiji 43[21]*)*

---

[20] Cases are generally presented in a way in which the gender of the patient cannot be discerned.
[21] 1910

Place of Origin: Saitama Prefecture

Family History: Father died of leprosy at the age of 43. Mother died of a certain uterine disease at the age of 30. No reports of any other relatives with leprosy. Other 6 siblings all healthy.

History of Leprosy: Patient healthy since infancy, not experiencing any significant illnesses. Was engaged in commerce before onset of leprosy, travelling widely for work. No history of measles, sensitivity to smallpox vaccine, or venereal disease. Whilst bathing one day at the age of 11, the patient noticed an area the size of a fingertip on the ulnar aspect of the right superior antebrachium in which a sensation of numbness could be felt, although this did not bother the patient. The patient's commercial activities continued until the age of 18, when bullae the size of one-sen copper coins formed at the left and right cubital joints. By the age of 21, areas of numbness were found on the buttocks and face, leading to the patient seeking medical attention. A diagnosis of "muscular rheumatism" was given. Officials conducting physical examinations at the time of conscription did not seem to consider this remarkable. Later, when the patient was bathing during the summer season, the patient's younger brother alerted the patient to a patch of discolouration in the patient's gluteal region. Thinking back on that day, although recalling that this patch was macular in appearance, the patient does not recall in which month of which year this occurred. At the age of 22, the hairs of the eyebrows began to fall out. Initially, the face was oedematous, assumed a dirty colour, and exhibited a certain sheen. It was at this point that the patient recognised it to be leprosy,[22] which resulted in the patient's fleeing. Around the age of 24 or 25, nasal discharge became foul-smelling, and lead to nasal congestion. Sometimes, due to the patient's scratching of this area, bleeding occurred; however, spontaneous nosebleeds were not been observed. Nodules formed but did not appear or protrude as elevations on the surface of the skin. It was also during this year that maculae appeared to spontaneously resolve. Since contracting leprosy, the patient has not experienced any febrile illnesses. At the age of 25, nodular-like eruptions appeared but subsided within a few days. Last February, the patient suffered from dry pleurisy of the right side.

Condition at the Time of Admittance: Constitution and nutrition moderate. Excepting the face and head, most hair had been lost. Face was a yellowish-brown complexion with a certain sheen. Infiltrations were mild. No eyebrows or eyelashes. Nose was flat, exhibiting a compacted

[22] The term used here is not the usual medical term used throughout this paper, but an archaic and colloquial term widely used in Japan at the time: 天刑病, which literally translates to "Heaven's Punishment Disease."

appearance. Semilunar infiltrations of sclera and upper navel area. Marked nystagmus, defects in the nasal septal cartilage, and left and right nasal cavities were perforated. Nasal mucosa was scarred, and deep inferior meatus was obstructed by blood clots. Limen nasi was eroded. Lips, oral cavity, and pharyngeal wall showed no abnormalities, though the left tonsil was mildly enlarged. No hoarseness of voice.

Extensor surfaces of the left and right upper limbs were dry and yellowish-brown; generally infiltrated. Several adzuki bean-sized subcutaneous nodules could be felt. Interosseous tendons of thenar and hypothenar eminences were atrophied. Claw-like appearance of middle finger observed.

Extensor surfaces of the lower limbs were, as with the upper limbs, dry and infiltrated. Hair remained only at the shins. Feet exhibited fallen arches with mild oedema.

Sensation was almost entirely lost, except for the medial surfaces of the upper and lower limbs. No severe trophic disturbances were observed. Hyperreflexia of the patellar reflex was observed.

Nerve thickening was not marked.
