# Supplementary material for: Descriptions of the natural history of erythema nodosum leprosum to inform clinical classification – A semi-systematic review
Source: PLoS Negl Trop Dis. 2026 Mar 23;20(3):e0014100. doi: 10.1371/journal.pntd.0014100 (PMC13035235; doi:10.1371/journal.pntd.0014100)
Supplement: Attachment — Submitted filename: PNTD-D-25-01473_response to reviewers.docx [file pntd.0014100.s004.docx]

**Manuscript ID:** PNTD-D-25-01473

**Title:** Descriptions of the natural history of erythema nodosum leprosum to inform clinical classification – a semi-systematic review

We thank the Editor and Reviewers for their careful review of our manuscript and for their constructive and insightful comments. Reviewer comments are reproduced in bold italics, followed by our responses.

### Reviewer #1: no comments to respond.

### Reviewer #2

### ABSTRACT

***Comment 1 (Methods, line 24):* The historical texts appear to have been included based on their significance rather than as a result of the systematic review process. We suggest clarifying this distinction.**

**Response:**

Thank you. We have clarified the selection of the inclusion of the historical texts by changing the sentence to:

“Historical texts published before 1940, prior to the introduction of sulfone antibiotics and corticosteroids, were purposively selected to capture descriptions of the natural history of ENL."

***Comment 2 (Results, line 33):*** **For greater clarity, it would be helpful to specify that the 41 studies refer to the contemporary literature.**

**Response:**
Thank you. We have addressed this point by rephrasing the sentence to:

“The systematic review, identified 572 articles after de-duplication, and 41 met inclusion criteria for providing definitions of ENL subtypes."

### AUTHOR SUMMARY

***Comment 3 (Lines 55–56):* It appears that other historical medical texts were available, but the five included were selected due to their importance. This selection criterion should be made explicit.**

**Response:**

Thank you. We have edited the paragraph referring to the texts as follows:

“We selected five historical medical texts published before antimicrobial and immunosuppressive treatments were available to identify descriptions of ENL and its natural history. We then compared them with modern published definitions of ENL identified following a systematic search of eight databases.”

***Comment 4 (Lines 65–66): Please review the sentence and replace one instance of the word “improve” to avoid repetition and enhance clarity.***

**Response:**
Thank you. We have edited the sentence which now reads:

“This would help improve the design of research studies for this challenging complication of leprosy.”

### KEYWORDS

***Comment 5:*** ***The keyword “leprosy” appears to be missing and should be included.***

**Response:**

Thank you. We have included ""leprosy” as a keyword along with “leprosy reactions” and “erythema nodosum leprosum"

### INTRODUCTION

***Comment 6 (Lines 114–115):*** ***Please review the sentence and replace or remove one instance of the word “conditions” to improve clarity.***

**Response:**
Thank you. We have rephrased the sentence as follows:

“However, there are no agreed definitions of classifying ENL with respect to chronicity and it is recognised that in many conditions there is a heterogeneous approach to classification according to duration^27^."

### METHODS

#### Narrative review

***Comment 7 (Lines 147–148):*** ***Please provide a clearer explanation of the rationale for including texts published prior to 1940, addressing both their historical relevance and the fact that the resolution of ENL symptoms may be influenced by antibiotic and immunosuppressive therapies, which were introduced after that period. At this stage of the manuscript, this selection criterion is not clear to the reader. The “inclusion criteria” later defined in the Methods section as a “temporal definition of ENL” does not appear to be appropriate for this selection.***

**Response:**
We have moved the text describing the rationale for choosing the five historical texts to the methods section. This now reads:

“We purposively selected five texts of leprosy published before 1940 prior to the use of sulfone antimicrobial therapy or corticosteroids for leprosy and leprosy reactions respectively^39,40^. The rationale for choosing these texts was that two are considered the seminal descriptive texts of early biomedicine^41,42^, one was the first to coin the phrase “erythema nodosum leprosum” (and subsequently considered an “excellent paper” despite not being widely cited)^43^, one has been cited as evidence that ENL is of short duration^34,35^ and one described ENL in the context of hydnocarpus oil treatment available prior to modern anti-microbial therapy^44^.”

The texts were reviewed for descriptions of ENL and its natural history.

***Comment 8 (Lines 206-207):*** ***The sentence included in the Results section (lines 206–207), “The rationale for choosing these texts was that two are considered the seminal descriptive texts of early biomedicine,” would be more appropriately presented as a methodological justification for this selection. As such, it appears better suited for inclusion in the Methods section rather than in the Results.***

**Response:**
Thank you. We believe that the explanation about which language version of texts was used is better left in the results section.

#### Systematic review

***Comment 9 (Lines 155–161):*** ***Considering the high specificity of the leprosy literature and the existence of two major international journals dedicated to this disease—Leprosy Review (first available issue in 1928) and the International Journal of Leprosy and Other Mycobacterial Diseases (first available issue in 1933), both of which provide advanced search tools (***[***http://leprev.ilsl.br/arquivo.php***](http://url.uk.m.mimecastprotect.com/s/QLOQCwr14SKk7pLF9i8HJwj_w?domain=leprev.ilsl.br) ***and*** [***http://ijl.ilsl.br/edicoes_anteriores.php***](http://url.uk.m.mimecastprotect.com/s/kJIhCx1g4ckDyOJIwsAHye6Xu?domain=ijl.ilsl.br)***) we recommend that these sources also be consulted to minimize the risk of missing relevant and important publications.
These sources could also be used to identify additional historical publications from the period preceding the introduction of antibiotics and corticosteroids for the treatment of leprosy reactions.***

**Response:**

We believe our search strategy was comprehensive as Leprosy Review is contained in MEDLINE/PubMed, LILACS, Scopus and EMBASE databases up to 2025, and the International Journal of Leprosy and Other Mycobacterial Diseases in PubMed, LILACS and Scopus up to 2005, including archival records and older content.

***Comment 10 (Lines 167–194):*** ***It should be noted that the sections included in this part of the manuscript (inclusion criteria, data management, selection process, and data collection process) appear to relate primarily to the systematic review rather than to the narrative review. For the sake of clarity and to facilitate reader understanding, it would be advisable to subdivide the Methods section into two clearly defined components: (1) Narrative Review and (2) Systematic Review. The methodological elements of the systematic review could then be presented as subsections (e.g., 2.1 Inclusion criteria, 2.2 Data management, etc.). Alternatively, the authors may consider an alternative structure that more clearly delineates these distinct methodological approaches.***

**Response:**

Thank you. We have capitalised the headings “Narrative Review” and “Systematic Review” to improve the clarity of the subdivisions employed.

### RESULTS

***Comment 11 (Table 1):*** ***Considering that not all texts published prior to 1940 were included, we suggest that the title of this table be revised to use “Selected texts.”***

***The third column (The description consistent with erythema nodosum leprosum) appears to be repetitive of the text that follows the table. The authors may wish to consider restricting the content of the table to the title, authors, year of publication, language, and the time course described, in order to improve the fluency and readability of the text.***

**Response:**
Thank you. We have edited the title as suggested. We believe the third column is needed to provide essential information to the reader.

***Comment 12 (Lines 229–236):*** **Clarification is needed regarding the use of the French original versus the English translation of Hansen and Looft.**

**Response:**
Thank you. The English translation of the original Norwegian text of Hansen and Looft is indicated in Table 1.

***Lines 229-236: In the Methods section, it is stated that the original French version of Hansen and Looft was used, as two of the co-authors are fluent French speakers. In this context, it is unclear why the same manuscript is also cited using the English translation by Professor Magnus Vollset. Was the clinical description compatible with ENL not already adequately presented in the original French version?***

***Furthermore, if the intention was to support the conclusion (possibly from Vollset) that the nodular form corresponds to the contemporary classification of lepromatous (LL) leprosy, we would recommend avoiding this approach, considering that such a conclusion cannot be reliably drawn from the historical descriptions alone, as borderline-lepromatous cases may present a similar clinical appearance and may also develop ENL*.**

Response:

Thank you. We have clarified earlier about the translated version of Hansen and Looft w used. The only French version reviewed by the authors was Danielssen and Boeck.

The English language version of Hansen and Looft was reviewed (see Table 1) by us. Hence having a native Norwegian speaker and recognised expert in the history of leprosy provide a modern interpretation of the original text is important. The point about the distinction between nodular forms of borderline lepromatous leprosy (far rarer) than nodular lepromatous forms in our opinion is moot as both borderline lepromatous leprosy and lepromatous leprosy are risk factors for erythema nodosum leprosum.

***Comment 13 (Table 2):*** ***In the first row (Type I), it is intriguing to note the description of fever, which would be more consistent with ENL. In the third row (Type III reaction), the description of a “reaction of old lesions” is more likely to correspond to a reversal reaction rather than ENL, although the authors may be referring to the worsening of old nodular lesions, corresponding to reactivation of subcutaneous nodules from previous episodes of ENL.
Therefore, in both instances, we would suggest that the authors exercise the same level of caution applied to the classification of Types VIII and IX in the Cullion classification and consider using the wording “Type 1 reaction and possibly some cases of ENL” for rows 1 and 3 (Types I and III).***

**Response:**

We believe our interpretation is cautious and are uncertain what the reviewer would prefer with respect to Types I and III. The Culion classification describes the Culion Type 1 pattern as: " Reaction of old lesions with or without fever". Our interpretation of this is that it is the “reaction of old lesions” which is the primary feature which occurs with or without fever. This is consistent with what today is recognised as a leprosy Type 1 reaction. We believe our suggestion of “Type 1 reaction and/or ENL” for Culion Type III is consistent with the view of the reviewer.

***Comment 14 (Lines 276–286): Repetitive citations from original texts hinder readability. Therefore, we recommend retaining only those excerpts that present divergent translations.***

**Response:**
Thank you. The subtle differences between the Portuguese and English versions of De Souza- Araújo’s treatise identified by our review is of potentially crucial importance in trying to divine the natural history of ENL described by De Souza- Araújo.

#### Systematic review – Results

***Comment 15 (Lines 301–304):*** ***As suggested in the comments about the Methods section for the systematic review, a simple search of abstracts using only the term “erythema nodosum leprosum” resulted in 5 publications prior to 1956, which may or may not cope with your inclusion criteria, but we suggest checking them:***

***- Volume: 19 - Edition: 3 - Jul/Aug/Sep - 1951
"Erythema nodosum" and "Erythema multiforme"
- Volume: 23 - Edition: 1 - Jan/Feb/Mar - 1955
The histopathology of acute panniculitis nodosa leprosa (erythema nodosum leprosum)
- Volume: 15 - Edition: 4 - Oct/Nov/Dec - 1947
Erythema nodosum in leprosy
- Volume: 14 - Edition: 1 - Jan/Dec - 1946
Erythema nodosum in leprosy. A study of the pathogenesis with reference to carbohydrate metabolism
- Volume: 5 - Edition: 4 - Oct/Nov/Dec - 1937
Erythema nodosum leproticum***

**Response:**

Thank you. We respectfully disagree. Four of the five works suggested were published after 1940, the cut-off we chose to ensure that sulfones and corticosteroids would not confound the interpretations of ENL.

The 1946 study by Pogge and Ross was conducted in individuals who had received sulfones and does not provide detailed description of the course of erythema nodosum leprosum.

The 1947 study by Wolcott uses data from individuals treated with sulfones. The 1951 short letter by Montel discussed the use of the terms erythema nodosum and erythema multiforme in leprosy but does not provide detailed clinical descriptions. The 1955 histopathology study by Pepler and colleagues does not add anything to the texts already used in our manuscript.

We have re-reviewed the1937 report of two cases by Reiss which while consistent with ENL was reported erythema nodosum complication leprosy and does not discuss the natural history.

***Comment 16 (Line 328):*** ***It would be preferable if the title of Table 3 followed the same format as those of Tables 4 and 5.***

**Response:**
Thanks you. The titles of the tables have been harmonised.

Table 3: Definitions of acute ENL in included studies with the number of subsequent citations

Table 4: Definitions of recurrent ENL in included studies with the number of subsequent citations

Table 5: Definitions of chronic ENL in included studies with the number of subsequent citations

Table 6: *Definitions of other temporal classifications of ENL in included studies with the number of subsequent citations*

***Comment 17 (Lines 331–333): Please review the statement that 116 cases (24.1%) “were diagnosed with ENL,” as it is unclear whether erythema nodosum leprosum was present at the time of leprosy diagnosis or whether these patients were diagnosed with ENL during the course of the cohort study.***

**Response:**
Thank you. The statement is accurate. The retrospective study reported 116 individuals with erythema nodosum leprosum, the timing of the onset of ENL would not affect this fact. We have not changed the text.

***Comment 18 (Line 366):*** ***It is noteworthy that this author (Mishra) used definitions that are peculiar and markedly different from those employed in all other studies. We believe that this discrepancy, and its comparison with other studies, should be highlighted in your Results section.***

**Response:**

Thank you. While we agree that the definitions used by Mishra are unusual, we highlight that “None of the studies proposing definitions for recurrent ENL in Table 4 provided clinical data to support the choice of interval between episodes of ENL. " It therefore seems difficult to justify singling out this particular study.

***Comment 19 (Table 6):*** ***If available, please expand the definition of acute recurrent ENL proposed by Pocaterra (2006) to facilitate a clearer understanding (first row).***

**Response:**
Thank you. The definitions “acute recurrent” and “acute multiple” used by Pocaterra are not always clearly delineated and we believe the Table 6 contains the best distillation of the definitions/descriptions used. We state that: “Pocaterra used the term “acute multiple ENL” interchangeably with “acute recurrent” episodes. These terms appear infrequently in the literature possibly resulting from lack of precision and potential redundancy within the classifications of ENL."

### DISCUSSION

***Comment 20 (Lines 401–412):*** ***As the main objective was to compare temporal classifications of ENL used in modern literature them with descriptions from the pre-corticosteroid era, it is important to recognize that no historical temporal classification of ENL was identified. Although the authors identified a historical classification used at Culion Hospital and adapted from de Souza-Araújo, this classification was based on clinical features only and did not include any temporal criteria.***

**Response:**

Thank you we have edited the introductory section of the discussion to include the following statement which accurately reflects our findings:

“Murata classified ENL as being “acute”, “subacute”, and “chronic” but neither he nor the authors of the other four texts selected reported the interval between episodes."

***Comment 21 (Line 410):*** **We suggest adding the word “spontaneous” before “symptoms resolution.”**

**Response:**

Thank you. We have added this important adjective.

***Comment 22 (Lines 450–453):*** ***Does acute ENL imply the presence of any interval between two or more episodes, given that it is defined as lasting less than six months? It is important to consider whether a patient with ENL could experience an initial episode, followed by regression, recurrence after the expected interval of 1–3 months, and subsequent regression without further flares, all within a total period of less than six months.***

***On this basis, it would be reasonable to interpret acute ENL as referring to a patient with a single episode of ENL lasting less than six months. If this rationale is accepted, no intervals between episodes would be expected. In this context, references to an “interval” would be appropriate only for recurrent or chronic ENL.***

**Response:**
Thank you. We have provided the data given in the publications and indicated to the reader that the definitions of acute ENL differ. Interpretation is challenging as alluded to because the studies are cross-sectional or retrospective. The reviewer makes good points about factors which need to be considered in definitions of acute ENL, and we believe we address this in our discussion of the term “"episode”.

***Comment 23 (Lines 472–473):*** ***We believe that the hypothesis that ENL could represent an adverse effect of therapy should not merely be mentioned in the Discussion but rather dismissed on the basis of your findings from the pre-sulfone descriptions.***

**Response:**

Thank you. We agree and make that very point.

***Comment 24 (General Discussion):*** ***In the Results section, it is stated that the first definition of acute ENL was published in 2006 by Pocaterra et al. (line 331); however, there is no indication of when the definitions of recurrent and chronic ENL were introduced. If this information was identified during your review, we believe it would be valuable to report and cite it.***

**Response:**

We have added sentences in the relevant sections.

“Kumar *et al*. published a retrospective study of leprosy reactions in Chandigarh, India which included the first definition of recurrent ENL"

“Okafor defined chronic ENL as more than three months which is the earliest definition of chronic ENL we identified.”

***Comment 25 (Lines 415–416):*** ***We suggest that this overlap, and the resulting difficulty in classifying certain patients, be explicitly acknowledged, as this represents a key conclusion of the manuscript. Highlighting this issue would strengthen the Discussion and support ongoing efforts to refine and harmonize ENL classification. Accordingly, the discussion of this topic could be expanded to remind readers of this overlap, noting that the definition of recurrent ENL includes periods without medication between episodes that may be as short as two weeks, whereas chronic ENL is described as having “near-continuous symptoms.”***

**Response:**
Thank you. We believe this is already clearly stated:

“However, definitions of recurrent and chronic ENL overlap substantially, with both categories referring to multiple or prolonged episodes following initial treatment.”

***Comment 26 (Lines 477–482):*** ***Lines 477-482: The notion of short survival among patients as a consequence of ENL should be interpreted with caution. According to Irgens et al. (Irgens LM, Nedrebø Y, Sandmo S, Skvennes A. Leprosy. Bergen, Norway: Leprosy Archives of Bergen; 2006), 86% of the 754 patients admitted to St. Jørgens Hospital in Bergen between 1822 and 1864 survived for up to 10 years after hospitalization. Although this cohort may have included cases of ENL, these findings more likely reflect the generally poor living and health conditions of the period. We believe that these contextual factors should be taken into account and discussed when interpreting these data.***

**Response:**
Thank you. We don’t believe that the quote used by us is at odds with the data provided by the reviewer.

“Intermittently all affections [fever and nodules] could spontaneously disappear and the patient would heal, but in general the life of the patient would end after eight or nine years”

We make no suggestion that survival is shortened but simply quote the text. This is important as a potential outcome of untreated ENL. There are data from the pre-multi-drug (rifampicin/dapsone/clofazimine) era of increased mortality associated with ENL.

BRUSCO CM, MASANTI JG. CAUSES OF DEATH OF LEPROSY PATIENTS, INFLUENCE OF LEPRA REACTIONS AND RENAL DISEASE. Int J Lepr. 1963 Jan-Mar;31:14-25. PMID: 14065224.

**Comment 27 (Lines 508–509): *It is clear that the manuscript identifies weaknesses in all current classifications of ENL. However, the authors’ proposed improvements are not clearly articulated, aside from their suggestion to conduct a Delphi study involving experts.***

**Response:**
Thank you. We have outlined clearly the deficiencies. These deficiencies result from researchers and clinicians in leprosy not having agreed definitions of temporal classifications of ENL based on good quality evidence. So rather than simply giving further opinion based, evidence-free assertions, we have elected to call for consensus based on good data.

Reviewer #3: No revisions suggested.

We hope that our responses and the revisions made satisfactorily address the comments raised by the Reviewer. We thank the Editor and Reviewers again for their time and thoughtful feedback, which have helped to improve the quality of this manuscript.

Kind regards,

**Dr Barbara de Barros**
On behalf of all co-authors
